# Design and selection of drug properties to increase the public health impact of next-generation seasonal malaria chemoprevention: a modelling study

**DOI:** 10.1016/S2214-109X(23)00550-8

**Published:** 2024-02-14

**Authors:** Lydia Braunack-Mayer, Josephine Malinga, Thiery Masserey, Narimane Nekkab, Swapnoleena Sen, David Schellenberg, André-Marie Tchouatieu, Sherrie L Kelly, Melissa A Penny

**Affiliations:** a Department of Epidemiology and Public Health, Swiss Tropical and Public Health Institute, Allschwil, Switzerland; b University of Basel, Basel, Switzerland; c Department of Disease Control, London School of Hygiene & Tropical Medicine, London, UK; d Medicines for Malaria Venture, Geneva, Switzerland; e Telethon Kids Institute, Nedlands, WA, Australia; f Centre for Child Health Research, The University of Western Australia, Perth, WA, Australia

## Abstract

**Background:**

Seasonal malaria chemoprevention (SMC) is recommended for disease control in settings with moderate to high *Plasmodium falciparum* transmission and currently depends on the administration of sulfadoxine–pyrimethamine plus amodiaquine. However, poor regimen adherence and the increased frequency of parasite mutations conferring sulfadoxine–pyrimethamine resistance might threaten the effectiveness of SMC. Guidance is needed to de-risk the development of drug compounds for malaria prevention. We aimed to provide guidance for the early prioritisation of new and alternative SMC drugs and their target product profiles.

**Methods:**

In this modelling study, we combined an individual-based malaria transmission model that has explicit parasite growth with drug pharmacokinetic and pharmacodynamic models. We modelled SMC drug attributes for several possible modes of action, linked to their potential public health impact. Global sensitivity analyses identified trade-offs between drug elimination half-life, maximum parasite killing effect, and SMC coverage, and optimisation identified minimum requirements to maximise malaria burden reductions.

**Findings:**

Model predictions show that preventing infection for the entire period between SMC cycles is more important than drug curative efficacy for clinical disease effectiveness outcomes, but similarly important for impact on prevalence. When children younger than 5 years receive four SMC cycles with high levels of coverage (ie, 69% of children receiving all cycles), drug candidates require a duration of protection half-life higher than 23 days (elimination half-life >10 days) to achieve reductions higher than 75% in clinical incidence and severe disease (measured over the intervention period in the target population, compared with no intervention across a range of modelled scenarios). High coverage is crucial to achieve these targets, requiring more than 60% of children to receive all SMC cycles and more than 90% of children to receive at least one cycle regardless of the protection duration of the drug.

**Interpretation:**

Although efficacy is crucial for malaria prevalence reductions, chemoprevention development should select drug candidates for their duration of protection to maximise burden reductions, with the duration half-life determining cycle timing. Explicitly designing or selecting drug properties to increase community uptake is paramount.

**Funding:**

Bill & Melinda Gates Foundation and the Swiss National Science Foundation.

## Introduction

Seasonal malaria chemoprevention (SMC) is recommended for *Plasmodium falciparum* malaria control in areas of seasonal transmission to reduce disease burden in children at high risk of severe malaria.^1^ SMC has so far depended on a complete treatment course of one dose of sulfadoxine–pyrimethamine plus three daily doses of amodiaquine, delivered at monthly intervals. Through these multiple deployments of an antimalarial drug, SMC maintains therapeutic drug concentrations during the high-risk period (ie, the high malaria transmission season). This intervention has been shown to be effective when delivered as part of routine malaria control, with an 88·2% (95% CI 78·7–93·4) mean reduction in the incidence of clinical malaria in children younger than 5 years within 28 days of administration of each cycle of SMC, and 42·4% (5·9–64·7) and 56·6% (28·9–73·5) mean reductions in malaria-related deaths in hospital in Burkina Faso and in The Gambia, respectively.^2^

The spread of partial *P falciparum* resistance to sulfadoxine–pyrimethamine might eventually threaten the effectiveness of SMC. Increasing frequency of the quintuple mutation associated with sulfadoxine–pyrimethamine resistance (ie, *pfdhfr*-51Ile, *pfdhfr*-59Arg, *pfdhfr*-108Asn, *pfdhps*-437Gly, and *pfdhps*-540Glu) has been observed in the sub-Sahel region.^2^ However, the overall frequency of the quintuple mutation and the frequency of mutations conferring partial resistance to amodiaquine (ie, *pfcrt*-CVIET, *pfmdr1*-86Tyr, and *pfmdr1*-184Tyr) remains low.^3^ Sulfadoxine–pyrimethamine resistance can reduce the duration of protection afforded by the drug in infancy and pregnancy,^4^, ^5^ and might negatively affect the provision of individual protection and population-level effectiveness currently associated with SMC.^6^, ^7^, ^8^ SMC appears to be maintaining its effectiveness as a public health control measure.^9^, ^10^ However, its effectiveness might not persist if the frequency of resistant mutations increases. More evidence is needed to assess this risk.

**Research in context**

**Evidence before this study**

We searched PubMed from database inception to Oct 14, 2022, without language restrictions for publications reporting product characteristics (ie, mechanism of action, preventive efficacy, and prophylaxis period) for candidate chemoprevention drugs and drug combinations, including atovaquone-proguanil with and without chloroquine, piperaquine, or pyronaridine; dihydroartemisinin and piperaquine with sulfadoxine–pyrimethamine; ganaplacide; MMV370; MMV731; and pyronaridine with amodiaquine, chloroquine, or piperaquine. Additionally, we searched PubMed for seasonal malaria chemoprevention (SMC) modelling studies using any drugs. The search terms were “((malaria[Title/Abstract]) AND (model*[Title/Abstract])) AND (seasonal malaria chemoprevention[Title/Abstract])”. Alternative medicines to SMC's standard of care (ie, sulfadoxine–pyrimethamine plus amodiaquine) are needed to safeguard the effectiveness of the intervention in reducing the burden of *Plasmodium falciparum* malaria from the potential threat of resistance and from poor adherence to the 3-day amodiaquine regimen. However, no published study has explored the importance of next-generation drug properties for the continued effectiveness of SMC. Little is known about how the drug properties contribute to the effectiveness of a chemoprevention programme, and about whether we can trade-off minimum product requirements against implementation regimens.

**Added value of this study**

By combining our previously established individual-based model with statistical analyses, we linked drug properties to public health impact for a range of chemoprevention drug profiles to define target product profile criteria for SMC. To inform candidate selection, we also did a validation analysis against clinical trial data of sulfadoxine–pyrimethamine plus amodiaquine for models of three different mechanisms of chemoprevention drug action, using detailed pharmacokinetic and pharmacodynamic models coupled with within-host parasite dynamics. By using multiple mathematical models that link drug properties to predicted public health impact of next-generation SMC, we offer quantitative, evidence-driven guidance for chemoprevention candidate selection. These results were substantiated over a range of modelled settings, providing guiding principles for chemoprevention candidate selection, development, deployment, and investment decisions.

**Implications of all the available evidence**

Our results indicate that, together with increasing intervention access and uptake, minimum criteria for duration of protection will drive the effectiveness of next-generation SMC. Selecting candidates and developing chemoprevention drugs with characteristics that favour high coverage, including a low number of doses, a non-inferior safety profile to sulfadoxine–pyrimethamine plus amodiaquine (including with repeated doses), and feasibility of a child-friendly formulation, will be key. Additionally, our results showed that the ideal chemoprevention drug profile is different from the ideal treatment profile, indicating the crucial importance of early preclinical and clinical evidence on duration of drug effectiveness for malaria control through pharmacokinetic and pharmacodynamic studies, malaria challenge trials, and early clinical studies.

To address the need for new SMC drugs, a clinical pipeline is being developed to repurpose existing drugs and to make new drugs available. Emerging guidelines have characterised target product profiles for chemoprevention interventions^11^ and, for the first time, funders and drug developers have targeted the development of candidates specifically for chemoprevention. Medicines for Malaria Venture (MMV)'s target product profile for chemoprotection^12^ underpins the research and development organisation's candidate pipeline, which includes MMV371 and ganaplacide. New SMC combination drug candidates have also been identified from a set of existing drugs used to treat clinical malaria or for prevention in travellers, such as atovaquone–proguanil,^13^ piperaquine, and pyronaridine.^11^

Yet in the early stages of product development, it remains unclear which drug characteristics will be the most important for the continued effectiveness of SMC. Emerging guidelines are benchmarking desired properties for drug candidates on the basis of the properties and protection afforded by sulfadoxine–pyrimethamine plus amodiaquine,^14^ without explicitly defining how these drugs can address the shortcomings of this standard of care (ie, limited intervention coverage, poor adherence to chemoprevention regimens, and negative side-effects or low tolerance). In particular, candidate selection will involve balancing desirable pharmacokinetic and pharmacodynamic properties with the delivery characteristics of SMC. However, to the best of our knowledge, no study has considered how the protection duration of a drug candidate, its protective efficacy, and implementation coverage can be balanced to increase the effectiveness of SMC. By protection duration, we refer to the period of time during which an individual is protected against infection after receiving a drug. By protective efficacy, we refer to a drug's within-host efficacy against one or more stages of the parasite lifecycle. This term is distinct from effectiveness, which refers to the clinical cases averted by deploying a drug in a population. By implementation coverage, we refer to the proportion of the target population for SMC with access to the intervention.

The impact of SMC with sulfadoxine–pyrimethamine plus amodiaquine beyond that observed in clinical trials has been shown in several observational studies and further explored in various studies with models built on a range of assumptions around the effects of the intervention on *P falciparum* blood or liver stages.^15^, ^16^, ^17^, ^18^ However, the mechanism of action for existing, alternative, and new SMC drugs has yet to be fully understood. Furthermore, no existing study has integrated uncertainty around the unknown pharmacological properties of next-generation drugs. Quantitative evidence that better accounts for these complexities is needed to define minimum product criteria for next-generation SMC and to increase the success rates for SMC drug development.

To address these knowledge gaps, we used mathematical and statistical modelling approaches to quantify minimum criteria for characterising the next generation of SMC drugs. We generated this evidence through engagement with malaria chemoprevention experts and through use of an individual-based malaria transmission model to analyse possible public health outcomes of three probable mechanisms of action for next-generation SMC drugs. Through this evidence, we aimed to provide funders and drug developers with guidance for the early prioritisation of new and alternative SMC drugs and their target product profile documents.

## Methods

### Expert consultation

This modelling study was grounded in engagement with chemoprevention drug and guideline developers as part of the June, 2021 convening Malaria Prevention: Shaping Next-Gen Medical Interventions.^19^ Consultations included representatives from 24 organisations globally, including international health organisations, funding organisations, product developers, and research institutes within malaria-endemic countries. Experts identified the need to quantify the impact of trade-offs between intervention characteristics (ie, efficacy, duration of protection, coverage, and dosing) on public health outcomes, and then set preferred ranges around these characteristics towards achieving health targets for next-generation SMC. These discussions shaped the use cases we considered and underpinned our approach to estimating the public health impact of SMC product candidates.

### Malaria transmission model

We applied an established individual-based malaria transmission model, OpenMalaria, to predict the impact of next-generation SMC products on population-level outcomes. The model and parameterisations used in this study have been fully described previously^20^, ^21^, ^22^ and are summarised in appendix 1 (pp 2–3). OpenMalaria consists of different model components representing the chain of processes from the mosquito lifecycle to malaria infection, treatment, and immunity acquisition of a human host, and captures differences in consequences of immunity on care seeking and clinical disease.^23^ The model variant^20^ used for this study includes a mechanistic within-host model component for the parasite lifecycle in humans, which describes the time course of asexual *P falciparum* parasitaemia after a single inoculation. Transmission from infected humans to mosquitoes depends on this asexual parasite density, with gametocyte densities emerging between 10 days and 20 days after infection.^22^, ^23^, ^24^ This model also incorporates pharmacokinetic and pharmacodynamic models for a comprehensive set of intervention dynamic characteristics.

The malaria transmission model was used to simulate several transmission and health system scenarios. Each scenario simulated a unique combination of setting characteristics (appendix 1 pp 3–4), including two levels of health system access to first-line malaria treatment (10% and 50%); two malaria transmission seasonality profiles (70% of cases occurring within a 3-month and 5-month period, respectively); and a range of transmission intensities, from low (8%) to high (39%) baseline annual prevalence of *P falciparum* infections detectable by rapid diagnostic tests in children aged 2–10 years under a no SMC counterfactual.

### Intervention dynamics and outcome measures

The use of models to infer a possible impact of a drug-based intervention relies on an accurate understanding of where and how the drug acts. However, for many SMC candidates, clinical evidence of drug activity and protective efficacy is not yet available. For this reason, we described the possible effect of an SMC candidate by using pharmacokinetic and pharmacodynamic models of three plausible mechanisms of action that describe the activity of a drug or drug combinations against blood-stage or liver-stage parasitaemia. For all three mechanisms, we represent the uncertainty inherent in modelling drugs whose pharmacokinetic and pharmacodynamic properties are not yet fully known by modelling a range of plausible drug properties and deployment coverage characteristics. Intervention modelling assumptions are fully described in appendix 1 (pp 4–5).

Our first approach to modelling next-generation SMC captured the effect of the intervention on reducing the growth rate of asexual blood-stage parasitaemia. We used a one-compartment pharmacokinetic and pharmacodynamic model to describe the parasite killing effect of a drug over time,^25^ referred to as SMC with blood-stage activity only. For simplicity, because pharmacokinetic and pharmacodynamic properties for next-generation SMC and pharmacodynamic properties for sulfadoxine–pyrimethamine are still unknown (unpublished), we assumed piperaquine-like behaviour and dosing.^26^ Second, we considered a candidate with both blood-stage and liver-stage activity by adding a model component that clears liver stage parasitaemia at the time of drug administration. This second model is referred to as SMC with dominant blood-stage activity and initial, complete liver-stage clearance. Finally, we considered candidates with predominantly liver-stage activity by deploying a previously calibrated model for sulfadoxine–pyrimethamine plus amodiaquine with protection against new infections that decays over time and with blood-stage and liver-stage parasite clearance at the time of drug administration.^27^ This model is referred to as SMC with dominant liver-stage activity and initial, complete blood-stage clearance. Unless specified, model properties and parameter ranges are the same across approaches (table 1).

**Table 1:** Summary of model characteristics and parameter ranges for each of the three mechanisms of action for an SMC candidate's effect

		**Parameter interpretation**	**Parameter value or range**
**Next-generation SMC with blood-stage activity only**			
Treatment schedule		Timing, delivery mode, and concentration at which the drug is given	Given orally at 0 h, 24 h, and 48 h once per SMC cycle
Pharmacokinetic parameters			
	Elimination half-life	Time for the drug concentration to fall by 50%	Sampled from 1 day to 20 days
	Volume of distribution	Theoretical drug volume needed to obtain observed blood plasma concentration levels	173 L/kg
Pharmacodynamic parameters			
	E_max_	Maximum rate at which the drug reduces parasite growth	Sampled from a rate of 2 to 30 per day
	EC_50_	Drug concentration at which 50% of E_max_occurs	0·0208 mg/L
	Slope	Controlling parameter for the shape of the concentration or effect decay (ie, the slope of the dose–response curve of a drug)	6
Round coverage (where one minus the round coverage is representative of the percentage of children who never receive a SMC dose)		Percentage of an eligible population randomly drawn to be included in each annual SMC round	Sampled from 70% to 100%
Cycle coverage		Within the sub-population receiving each annual SMC round, the percentage of children randomly drawn to receive each cycle of SMC	Sampled from 70% to 100%*
**Next-generation SMC with dominant blood-stage activity and initial, complete liver-stage clearance**			
Treatment schedule		Timing, delivery mode, and concentration at which the drug is given	Blood-stage drug given orally at 0 h, 24 h, and 48 h once per SMC deployment at 18 mg/kg; liver-stage clearance drug given orally at 0 h once per SMC deployment
**Next-generation SMC with dominant liver-stage activity and initial, complete blood-stage clearance**			
Treatment schedule		Timing, delivery mode, and concentration at which the drug is given	Blood-stage clearance drug given orally at 0 h once per SMC deployment; liver-stage clearance drug given orally at 0 h once per SMC deployment
Decay profile		Shape of the decrease in protective efficacy of a drug over time from its initial efficacy	Weibull function with shape parameter k=5·34 and scale parameter representing the duration of protection half-life
Duration of protection half-life		Number of days until the protective efficacy of a drug decays to 50% of its maximum effect	Sampled from 10 days to 60 days
Initial efficacy		Probability of preventing individual infection at the time of administration	Sampled from 80% to 100%

SMC=seasonal malaria chemoprevention.

* The combined values of round and cycle coverage determine both the proportion of children who receive at least one SMC cycle and who receive all SMC cycles in a given round. If round and cycle coverage vary between 70% and 100%, then between 70% and 100% of children can receive at least one SMC cycle, and between 17% and 100% of children can receive all SMC cycles.

This complex modelling approach was not intended to be used to investigate the effect of drug activity on the effectiveness of SMC, but rather was necessary to capture a broad spectrum of drug properties across the entire chemoprevention candidate development pipeline. By doing so, we calculated desired ranges for intervention coverage and drug initial efficacy and protection duration to inform an understanding of candidate impact.

We explored the importance of next-generation SMC drug characteristics for different deployments by simulating outcomes for three, four, and five cycles of SMC delivered to children aged 3–59 months. In line with WHO terminology, we used cycle to refer to the monthly administrations of SMC in a given year and round to refer to each annual deployment of an SMC programme. For deployments with three and four cycles, the first cycle was deployed in the month before the seasonal peak. For five cycles, the first cycle began 2 months before the seasonal peak (appendix 1 p 4). We also modelled SMC using the same aforementioned criteria for a broader target population—ie, children aged 3–119 months. Primary outcome measures were the reduction in clinical incidence, prevalence, and severe disease incidence in the target populations during the 3-month, 4-month, and 5-month intervention periods 5 years after the first deployment of SMC. Reductions were calculated relative to a no SMC counterfactual measured in the same period and target populations (children aged 3–59 months) from the year before the introduction of SMC. All outcome measures and deployment characteristics are fully described in appendix 1 (pp 3–4).

### Standard-of-care parameters

Clinical evidence of drug activity, duration, and protective efficacy is not yet available for many SMC candidates. For this reason, in the absence of data for model validation, we assessed the ability of our three drug models to accurately represent the dynamics of sulfadoxine–pyrimethamine plus amodiaquine, the standard of care of SMC. We did this analysis by replicating protective efficacy outcomes from a randomised non-inferiority trial of dihydroartemisinin–piperaquine to sulfadoxine–pyrimethamine plus amodiaquine,^26^ following the approach described by Burgert and colleagues.^27^ The protective efficacy of sulfadoxine–pyrimethamine plus amodiaquine against clinical case incidence was extracted from the findings published by Zongo and colleagues.^26^ For each SMC modelling approach (ie, blood-stage activity only, dominant blood-stage activity, and dominant liver-stage activity), we used OpenMalaria to perform in-silico clinical trials for multiple samples from model parameter values. We then identified the range of parameter values that produced similar protective efficacy to sulfadoxine–pyrimethamine plus amodiaquine by minimising the residual sum of squares between simulated trial outcomes and the protective efficacy of sulfadoxine–pyrimethamine plus amodiaquine. The approach is fully described in appendix 1 (pp 4–6).

### Simulation and statistical analysis

We applied a mathematical framework for predicting determinants of intervention impact and defining minimum drug profile criteria. This framework has been previously described,^28^ and was used in a proof-of-concept study^27^ (appendix 1 pp 6–7). Briefly, we used the individual-based malaria transmission model to simulate scenarios over many input values for intervention coverage and drug initial efficacy and protection duration. Heteroskedastic Gaussian process regression models were then trained to capture the association between intervention inputs and public health outcomes. These machine learning models permitted us to explore a large parameter space with fewer calls to the computationally intensive simulation model. Using these model emulators, we did a global sensitivity analysis using the Sobol-Jansen method^29^ to identify synergisms between intervention characteristics. We also used optimisation methods to identify minimum drug criteria towards achieving a target outcome.

### Role of the funding source

Together with other experts, representatives from the Bill & Melinda Gates Foundation contributed to the discussions regarding SMC use cases. The funders of the study had no role in study design, data simulation, data analysis, data interpretation, decision to publish, or writing of the report.

## Results

**Figure 1:**
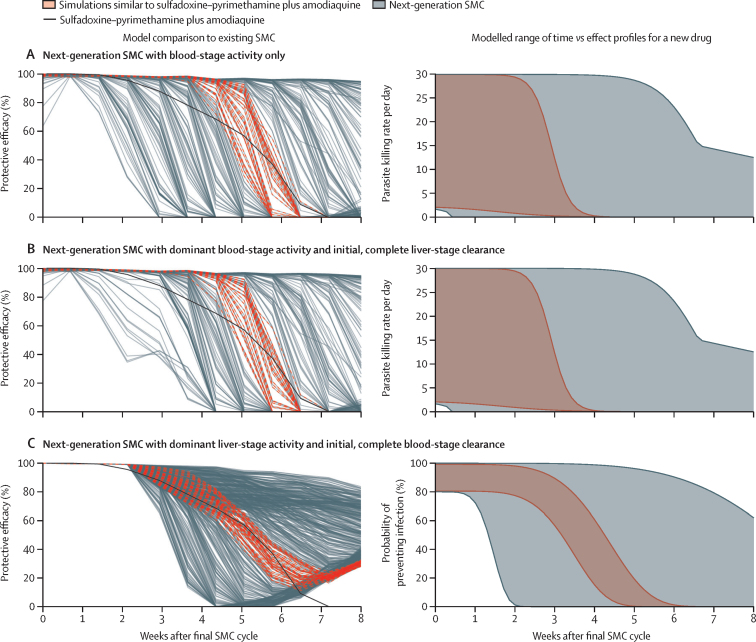
Model comparison with the protective efficacy of sulfadoxine–pyrimethamine plus amodiaquine for each of three SMC modelling approaches Left figure panels show the protective efficacy of in-silico clinical trial outcomes for each simulated next-generation SMC profile. Grey curves indicate the model-estimated protective efficacy against clinical case incidence of each of 500 simulated SMC drug profiles. Solid black curves show the protective efficacy of sulfadoxine–pyrimethamine plus amodiaquine against clinical case incidence, as extracted from data published by Zongo and colleagues.^26^ Red dashed curves indicate a selection of the 500 simulated SMC drug profiles with a similar model-estimated protective efficacy to sulfadoxine–pyrimethamine plus amodiaquine. This selection was assessed by calculating the residual sum of squares between simulated trial outcomes and the protective efficacy of sulfadoxine–pyrimethamine plus amodiaquine and by identifying the simulated profiles with a residual sum of squares within 0·1 SD of the minimum residual sum of squares. Right figure panels show the full parameter space of modelled time–effect relationships for a new drug (grey shaded regions). The red shaded regions indicate the space of parameter profiles with a similar model-estimated protective efficacy to sulfadoxine–pyrimethamine plus amodiaquine, as indicated in the left figure panels. The dominant liver-stage activity model has a different model structure compared with the other two models, and therefore its parameters are different. SMC=seasonal malaria chemoprevention.

For SMC with blood-stage activity only and dominant blood-stage activity, we found that an elimination half-life of 5–9 days for both models produced behaviours similar to sulfadoxine–pyrimethamine plus amodiaquine—a range similar to published studies investigating the pharmacokinetics of sulfadoxine–pyrimethamine.^30^ For SMC with dominant liver-stage activity, we estimated that a drug with a duration of protection half-life (representing the time until the protective efficacy of a drug reaches 50% of its initial value, a different metric from elimination half-life) of 24–30 days would have a similar protective efficacy to sulfadoxine–pyrimethamine plus amodiaquine (figure 1, table 2).

**Table 2:** Parameter ranges of the SMC model with a similar protective efficacy to sulfadoxine–pyrimethamine plus amodiaquine

	**Modelled range**	**Range with a similar protective efficacy to sulfadoxine–pyrimethamine plus amodiaquine**
**SMC with blood-stage activity only**		
Elimination half-life, days	1–20	5–9
Duration of protection half-life, days	4–46	11–20
E_max_	2–30	2–30
Slope	1–8	1–8
**SMC with dominant blood-stage activity and initial, complete liver-stage clearance**		
Elimination half-life, days	1–20	5–9
Duration of protection half-life, days	4–46	11–20
E_max_	2–30	2–30
Slope	1–8	1–8
SMC with dominant liver-stage activity and initial, complete blood-stage clearance*		
Initial efficacy, %	80–100%	81–99%
Duration of protection half-life, days	10–60	24–30

Data are range (minimum–maximum). The protective efficacy for each parameter was considered to be sufficiently close to that of sulfadoxine–pyrimethamine plus amodiaquine if the residual sum of squares was within 0·1 SD of the minimum residual sum of squares. The protective efficacy of sulfadoxine–pyrimethamine plus amodiaquine against clinical case incidence was extracted from data published by Zongo and collagues.^26^ E_max_=maximum parasite killing rate per day. SMC=seasonal malaria chemoprevention.

* The dominant liver-stage activity model has a different model structure compared with the other two models, and therefore its parameters are different.

**Figure 2:**
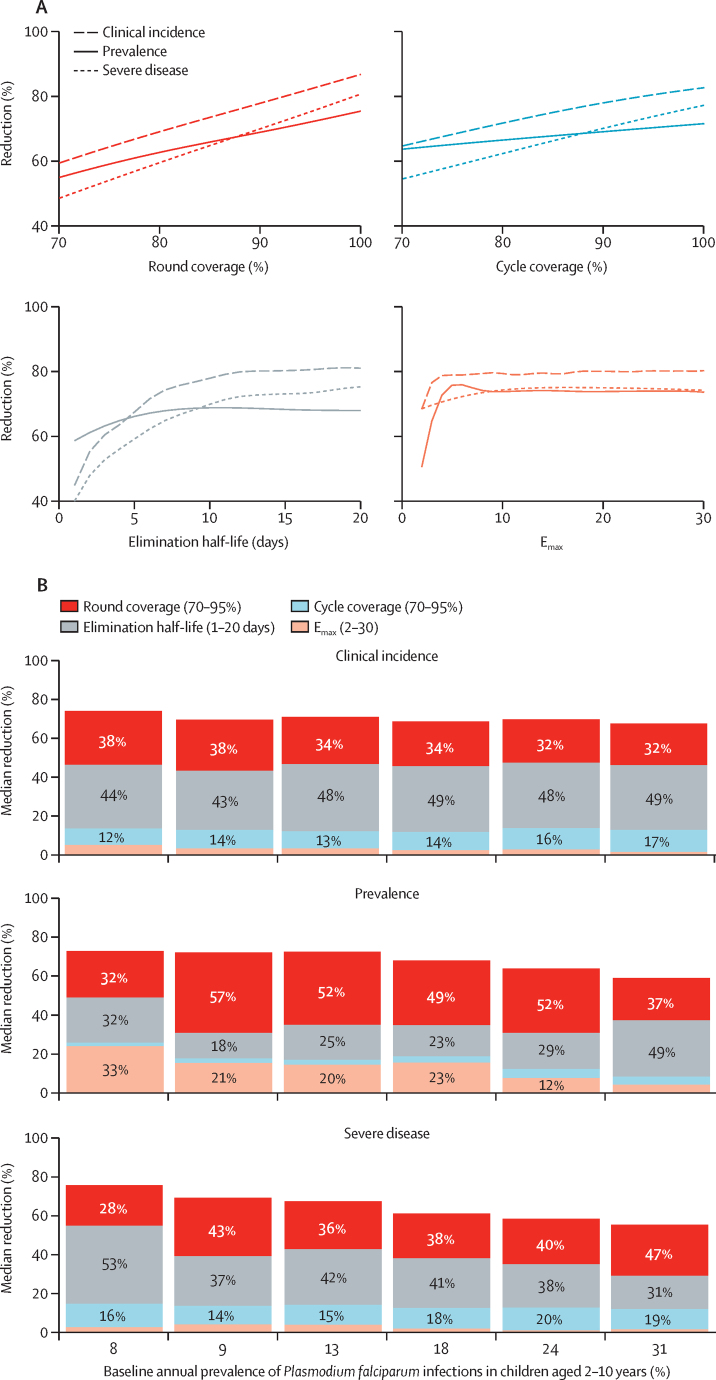
Emulator predictions and drivers of impact for relationships between SMC properties and clinical incidence, prevalence, and severe disease reductions, for SMC with dominant blood-stage activity and initial, complete liver-stage clearance (A) Gaussian process regression emulator predictions. Results show a scenario with 50% access to first-line treatment during 14 days and a 5-month seasonal profile with a baseline annual prevalence of *Plasmodium falciparum* infections in children aged 2–10 years of 18% when SMC was deployed in four monthly cycles to children aged 3–59 months in a given year. Each panel shows the predicted reductions in clinical incidence, prevalence, and severe disease when all SMC performance characteristics but the parameter of interest were held constant at 90% for round coverage, 90% for cycle coverage, 10 days for elimination half-life, and 3·45 for E_max_. Alternative profiles for these constant parameters are shown in appendix 1 (p 16). (B) Drivers of impact on predicted clinical incidence, prevalence, and severe disease reduction for SMC, compared with a no intervention counterfactual. Bars indicate the total Sobol-Jansen effect indices for key model parameters. Indices can be interpreted as the proportion of variation in the outcome attributable to a given change in each variable, along with its interactions with other variables. Bar heights indicate the median expected reduction across the modelled parameter range. Results are shown across prevalence settings for a scenario of 50% access to first-line treatment within 14 days with a 5-month seasonal profile, where SMC was deployed in four monthly intervals in a given year to children aged 3–59 months. Percentage values less than 8% are not shown. E_max_=maximum parasite killing rate per day. SMC=seasonal malaria chemoprevention.

For all three drug models and across the range of intervention properties evaluated in this study, our analysis showed that the protection duration of an SMC candidate is crucial for minimising malaria morbidity. For SMC with blood-stage activity only, drug elimination half-life was the most important driver of impact on clinical incidence, prevalence, and severe disease reduction across the majority of the modelled scenarios (appendix 1 pp 8, 10, 13). Elimination half-life was the most or second-most important driver for SMC with dominant blood-stage activity (figure 2; appendix 1 pp 11, 14). For example, when SMC with dominant blood-stage activity was deployed to children aged 3–59 months, the elimination half-life explained up to 54% of variation across all outcome measures and across all modelled scenarios. Duration of protection half-life for SMC with dominant liver-stage activity had a lesser importance (appendix 1 pp 9, 12, 15), driven by the model assumption of blood-stage and liver-stage parasite clearance at the time of administration.

Our modelling suggests that the initial parasite killing effect of an intervention was important to reduce malaria prevalence in the target population. In our models, the parasite killing effect can be thought of as capturing the impact of adherence to a drug's dosing schedule or of the drug's initial treatment efficacy (appendix 1 p 4). The rate of blood-stage parasite clearance by a drug (ie, the maximum parasite killing rate per day [E_max_]) accounted for up to 33% of variation in prevalence reduction across different values of baseline annual prevalence of *P falciparum* infections in children aged 2–10 years (figure 2B). The importance of the parasite killing effect of a drug suggested that prevalence reduction was driven by both the effectiveness with which a drug cleared blood-stage parasites and the duration over which the drug prevented new infections from taking hold. Drug elimination half-life remains more important than the parasite killing effect for impact on clinical disease. The ability of our individual-based model to capture differences in immunity means that clinical incidence and prevalence endpoints provide a mechanism to translate across different settings and, thus, can facilitate the benchmarking of endpoints between different clinical trials and SMC candidates.

In addition to a drug's elimination half-life and blood-stage activity, SMC coverage was a crucial determinant of public health impact. Our sensitivity analysis of results for SMC with dominant blood-stage activity indicated that, for a scenario with 50% access to first-line treatment and a 5-month seasonal profile, between 35% and 69% of variation in clinical incidence, prevalence, and severe disease reduction could be attributed to the combined impact of SMC's round coverage (ie, the proportion of children with access to an SMC round, meaning to the three, four, or five cycles of an annual SMC campaign) and cycle coverage (ie, the proportion of children with access to a round who also receive each SMC cycle; figure 2B). As both round and cycle coverage increased, SMC was predicted to reduce clinical incidence, prevalence, and severe disease in children who receive the intervention (figure 2A). Coverage was similarly crucial for the burden reduction of SMC modelled as dominant liver-stage activity, but was less important than the elimination half-life when SMC was modelled with blood-stage activity only, driven by the fact that drug duration makes up for the lack of complete liver-stage parasite clearance at the time of drug administration in this model (appendix 1 pp 8–9).

SMC coverage and elimination half-life were the key impact drivers for next-generation SMC across seasonal profiles (3-month and 5-month) and across levels of access (10% and 50%) to first-line curative treatment for malaria (appendix 1 pp 13–15). Similar results were observed when SMC was deployed to children aged 3–119 months (appendix 1 pp 10–12). We also observed evidence of very low indirect benefits of SMC on children aged up to 119 months and not receiving SMC, particularly for drug candidates with high coverage and long elimination half-life (appendix 1 p 17).

**Figure 3:**
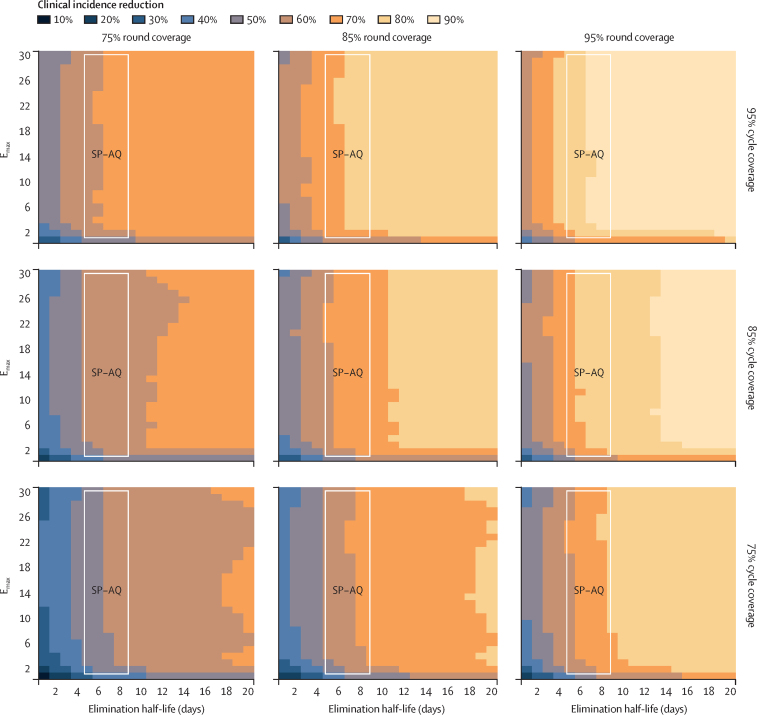
Impact of a change in coverage on the predicted relative reduction in clinical incidence (measured over a 4-month intervention period) after SMC compared with a no intervention counterfactual, for SMC with dominant blood-stage activity and initial, complete liver-stage clearance Each square in the grid indicates the predicted reduction (rounded to the nearest 10%) if an intervention with the given elimination half-life and E_max_ were deployed, assuming a slope of 6. Variation in this figure is driven by the combined effect of stochastic uncertainty and emulator prediction error. Results are shown for children aged 3–59 months for a 5-month seasonal profile with a baseline annual prevalence of *Plasmodium falciparum* infections in children aged 2–10 years of 18%, where access to first-line treatment was 50% within 14 days and where SMC was deployed four times at monthly intervals in a given year surrounding peak seasonality. Each panel represents results for a different level of SMC round coverage (75%, 85%, and 95%) and cycle coverage (75%, 85%, and 95%). The white region indicates the space of parameter profiles with a similar protective efficacy to sulfadoxine–pyrimethamine plus amodiaquine (ie, residual sum of squares for in-silico protective efficacy compared with the protective efficacy of sulfadoxine–pyrimethamine plus amodiaquine falling within 0·1 SD of the minimum residual sum of squares). E_max_=maximum parasite killing rate per day. SMC=seasonal malaria chemoprevention. SP–AQ=sulfadoxine–pyrimethamine plus amodiaquine.

Our results highlight a potential to trade a reduced dosing schedule and, hence, potentially shorter elimination half-life in favour of facilitating higher SMC coverage for impact on clinical incidence and severe disease. In moderate transmission intensities with a baseline annual prevalence of *P falciparum* infections in children aged 2–10 years of 18%, where SMC was deployed with four monthly cycles annually to children aged 3–59 months, an intervention with an elimination half-life of 10 days and E_max_ of 10 was predicted to achieve a 74% reduction in clinical incidence (95% prediction interval 67–80%) when both round and cycle coverage were 85% (figure 3). Deploying the same intervention with 95% round coverage led to an increase in the expected clinical incidence reduction to 83% (95% prediction interval 79–87%). Similar trade-offs were apparent for SMC with blood-stage only activity (appendix 1 p 18) and dominant liver-stage activity (appendix 1 p 19).

**Figure 4:**
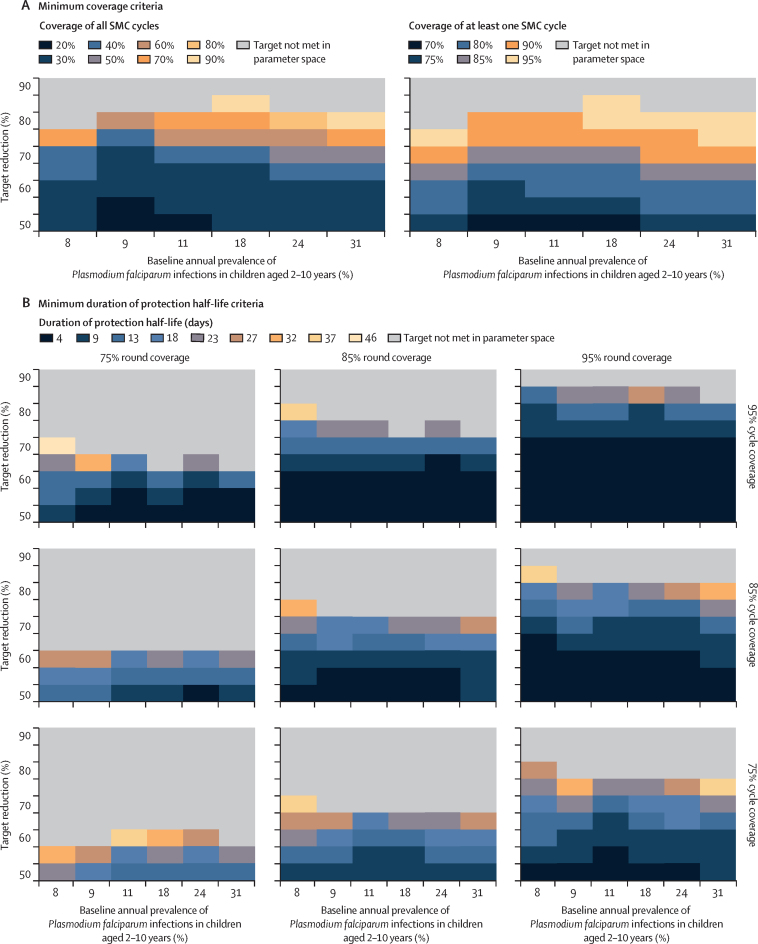
Summary of predicted minimum parameter values for SMC with dominant blood-stage activity and initial, complete liver-stage clearance to achieve target reductions in clinical incidence and severe disease (A) Summary of the predicted minimum coverage criteria for SMC towards achieving target reductions in clinical incidence and severe disease across SMC deployments, compared with a no intervention counterfactual. Results show estimated minimum values for SMC coverage characteristics whose 95% prediction interval was higher than the given target reduction for both clinical incidence and severe disease (vertical axis), aggregated across outcomes (clinical incidence and severe disease reductions measured across the intervention period), SMC deployments (three, four, and five monthly cycles of SMC in a given year), and levels for other model parameters (elimination half-life and E_max_). Results are shown for a 5-month seasonal profile in a setting with 50% access to first-line treatment, where SMC was deployed to children aged 3–59 months. (B) Summary of the predicted minimum duration of protection half-life criteria for SMC towards achieving a target reduction in clinical incidence and severe disease for varying levels of SMC round and cycle coverage, shown for a 5-month seasonal profile in a setting with 50% access to first-line treatment, where SMC was deployed in four monthly cycles to children aged 3–59 months. Results show the estimated minimum duration of protection half-life with a 95% prediction interval higher than the given target reduction in clinical incidence (vertical axis), compared with a no intervention counterfactual and measured across the 4-month intervention period. Duration of protection half-life is defined as the number of days until the parasite killing effect of a drug reaches 50% of its maximum effect. Minimum criteria were calculated across E_max_ levels. E_max_=maximum parasite killing rate per day. SMC=seasonal malaria chemoprevention.

Together, these results indicate that, for increased public health impact, SMC requires an intervention with an extended duration of protection between cycles, and with high coverage. After an optimisation procedure (appendix 1 p 6), we identified minimum requirements for these key intervention characteristics across modelled scenarios for SMC with dominant blood-stage activity. For coverage of SMC, our results indicate that more than 60% of children should receive all cycles and more than 90% should receive at least one cycle of SMC to achieve targets of 75% clinical incidence and severe disease reduction across transmission settings (figure 4A). Extended protection between SMC cycles is also required. For example, for a scenario where SMC was delivered in four monthly cycles to children aged 3–59 months with high coverage (85% round coverage and 95% cycle coverage, translating to 69% of children receiving all SMC cycles), a duration of protection half-life of more than 23 days was required to achieve a 75% clinical incidence and severe disease reduction (figure 4B). This duration half-life is longer than the duration half-life we estimated for sulfadoxine–pyrimethamine plus amodiaquine in our model validation exercise, suggesting scope to further optimise SMC with sulfadoxine–pyrimethamine plus amodiaquine by shortening the time between cycles.

## Discussion

We used an individual-based model of malaria transmission combined with multiple mechanisms of action and a broad range of drug properties, validated with clinical trial data for sulfadoxine–pyrimethamine plus amodiaquine, to link individual-level drug characteristics to the possible population-level impact of new drugs for SMC. We identified which drug and intervention characteristics were the most important contributors to the impact of SMC on public health. We found that impact on clinical incidence was primarily determined by the campaign coverage of SMC and by the protection duration of a drug. More specifically, for spacing of 4 weeks between SMC cycles, a protection duration longer than that currently provided by sulfadoxine–pyrimethamine plus amodiaquine was required to minimise clinical incidence and maximise prevalence reductions. If, however, we aimed to reduce prevalence, then high initial efficacy was also essential. Importantly, these findings quantitatively show that the ideal drug profile for malaria treatment is different from the ideal chemoprevention profile; the ideal drug profile requires high efficacy and does not require long protection duration, whereas the ideal chemoprevention profile can have lower efficacy but must have a long duration.

Our findings quantify the intuitive notion that protection duration of a chemoprevention candidate is essential for determining which candidates are likely to have the greatest effectiveness beyond clinical trials. By modelling three, four, and five cycles of SMC, we evaluated how the ideal drug duration might change if the deployment of SMC does not cover the full malaria transmission season. Varying cycle timing highlighted that, to minimise clinical incidence and maximise prevalence reductions, the length of time between SMC cycles should be matched to the protection duration of the drug. Furthermore, in our blood-stage models, variation in a candidate's duration of protection was captured by varying the drug's elimination half-life. For a real drug candidate, the protection duration of a drug will be determined by the interplay between pharmacokinetic and pharmacodynamic characteristics, including its half maximal effective concentration, minimal effective concentration, and effect decay profile. For drug combinations, protection duration will be further influenced by the presence of drug–drug interactions. The precise balance of potency, pharmacokinetics, and host interactions required to maximise duration will be specific to each product, and these properties will need to be determined by drug developers for each chemoprevention candidate.

The importance of protection duration for a chemoprevention drug substantiates the necessity of routine monitoring of chemoprevention efficacy against any infection endpoints, which should be measured in both early-stage and late-stage clinical studies of pharmacokinetic and pharmacodynamic properties, and when chemoprevention is routinely deployed.^31^ Ideally, this monitoring against any infection endpoints would be supported through human challenge studies of drug activity against both blood-stage and liver-stage parasites, such as for the recently completed trial for cabamiquine.^32^ Natural infection phase 2 studies with highly sensitive PCR endpoints can also be used. Published pharmacokinetic and pharmacodynamic evidence from clinical trials is available for some combination SMC candidates, such as for atovaquone–proguanil plus amodiaquine.^13^ This combination showed unanticipated extrapyramidal effects,^13^ reinforcing the need for rigorous assessment of repurposed drugs when used in combination. However, to the best of our knowledge, no published clinical trial has looked for synergies between pharmacokinetic and pharmacodynamic properties of other combination SMC candidates, including atovaquone–proguanil plus chloroquine, plus piperaquine, or plus pyronaridine; as a result, evidence of the effect of such synergies on protection duration of an SMC candidate is scarce. At the time of writing, two ongoing trials (NCT03726593 and NCT05689047) are assessing efficacy outcomes for some of these chemoprevention combination candidates.

Regardless of protection duration of SMC candidates, the malaria product development community needs to reach consensus on the importance of drug activity for chemoprevention impact to make informed drug selection decisions. Crucially, the clinical trial evidence so far described is not available for sulfadoxine–pyrimethamine with or without amodiaquine, and our field's knowledge of sulfadoxine–pyrimethamine's action against liver-stage parasitaemia is based on studies of pyrimethamine alone.^33^ In particular, evidence indicates that sulfadoxine–pyrimethamine plus amodiaquine retains its effectiveness for chemoprevention in geographical regions with moderate prevalence of the *dhfr/dhps* quadruple mutant (ie, *pfdhfr*-51Ile, *pfdhfr*-59Arg, *pfdhfr*-108Asn, and *pfdhps*-437Gly) associated with sulfadoxine–pyrimethamine treatment failure.^9^, ^10^ Yet our lack of understanding of the liver-stage action of sulfadoxine–pyrimethamine plus amodiaquine means that the reasons for its continued effectiveness remain unclear. Furthermore, part of the effectiveness of sulfadoxine–pyrimethamine in other chemoprevention programmes, such as intermittent preventive therapy in pregnancy, might be due to non-malarial pathways.^34^ Because of the difficulty and cost of running clinical trials and observational studies, mathematical modelling has an important role in building consensus on drug activity. Further modelling studies could use scenario-based modelling with pharmacokinetic and pharmacodynamic models to develop hypotheses for the contribution of SMC to immunity acquisition, effectiveness against resistant parasites, and non-malarial pathways.

Our results indicate that the parasite killing effect of a chemoprevention drug is of lower importance for its public health impact on averting clinical and severe disease than its elimination half-life. This finding indicates that the ideal chemoprevention drug profile is different from the ideal treatment profile, which must have high efficacy to clear parasites. The protection duration of a drug is likely to have long-term effects on immunity acquisition for SMC's target population—that is, children who might have low parasitaemia but who do not present with clinical malaria. A chemoprevention drug that prevents clinical symptoms but does not rapidly clear parasitaemia might allow children to build malaria immunity. Importantly, this possibility might justify repurposing antimalarial drugs that did not meet minimum treatment efficacy thresholds.

However, repurposing an antimalarial drug that does not offer very high efficacy but has a long duration of protection does raise several considerations. First, as a field, we must consider that permitting low-grade parasitaemia might have a negative impact on a child's health. Second, by deploying a chemoprevention product that does not rapidly clear parasitaemia, the risk of parasite resistance may increase. This risk could, in turn, reduce the usable lifespan of a chemoprevention product, and it could also require that the repurposed drug is deployed in combination with another antimalarial drug with similar pharmacokinetic properties, such as the elimination half-life, to avoid resistance selection. These considerations must be balanced against the crucial need for an alternative to sulfadoxine–pyrimethamine plus amodiaquine for chemoprevention interventions.

In addition to indicating that intervention coverage is crucial for the public health impact of SMC, our results suggest that optimising drug properties likely to affect the coverage of SMC will be more important than optimising duration of protection to improve population impact beyond what is currently observed with sulfadoxine–pyrimethamine plus amodiaquine. Drug dosing frequency and concentration is typically chosen to maximise the effect of a drug while maintaining an acceptable safety profile and allowing for sufficient adherence. For SMC candidate selection, however, a product with a reduced protection duration might be preferred in favour of increased adherence. Safety on repeated dosing is of particular concern for drug use in repeated chemoprevention cycles, potentially requiring lower dosage than for other use cases. Furthermore, parental reluctance to allow a child to receive any drug has been identified as a major hurdle to adherence with sulfadoxine–pyrimethamine plus amodiaquine,^35^ and the three-dose schedule and known side-effects of nausea and vomiting associated with sulfadoxine–pyrimethamine plus amodiaquine are likely to be substantial drivers of non-adherence. Increased effectiveness could be achieved by providing an intervention with the least frequent dosing regimen possible to achieve a minimum acceptable efficacy. For SMC and for other chemoprevention programmes, such as perennial malaria chemoprevention, child-friendly formulations should be pursued as early as possible, as has been done for sulfadoxine–pyrimethamine. Close collaboration between drug developers and regulatory bodies will be crucial for integrating early feedback on the appropriate balance between drug efficacy and campaign coverage.

Development pathways for new chemoprevention drugs also need to be designed to engage communities early and frequently to secure buy-in and to address barriers to coverage early on. Questions regarding optimal dosing and cycle frequency should be explored before phase 2 trials in collaboration with malaria control programmes, to ensure that the drug regimens evaluated can then be feasibly deployed within a malaria control programme. Patient-reported outcome measures have also become widely accepted measures of treatment benefit and risk in therapeutic areas such oncology and ophthalmology, where medical treatment focuses on extending or improving quality of life.^36^ The inclusion of patient-reported outcome measures in phase 2 and 3 chemoprevention trials could allow for community preferences for drug deployment to be identified before the pilot studies, enabling selection of dosing regimens most likely to maximise drug adherence and achieve adequate SMC coverage.

As in any modelling study, this study has some limitations. First, our three-drug models might not capture the as-yet-unknown dynamics of new chemoprevention drugs. In particular, both dominant blood-stage and liver-stage models assume complete liver-stage and blood-stage parasite clearance, respectively, at the time of administration. This assumption could underplay the importance of the parasite killing effect of a drug and should be reconsidered as new clinical evidence becomes available.

Second, our three-drug models described varying mechanisms of action for one drug with a rapid decay in protective effect, which might not accurately represent drug combination candidates. In particular, our one-compartment pharmacokinetic and pharmacodynamic models cannot fully represent drug–drug interactions and, crucially, they are unable to capture the importance of combining drug candidates with matching half-lives to prevent parasite resistance. However, representing the combined action of multiple drugs with a single model offers a balance between model complexity and the substantial unknowns regarding chemoprevention drug pharmacokinetic and pharmacodynamic properties. In the future, as more clinical evidence for chemoprevention candidates becomes available, multiple models might be necessary to capture the effects of drug synergisms and interactions.

Finally, although we included pharmacokinetic and pharmacodynamic models within our large population model, our estimates for the effectiveness of next-generation SMC were based on a single, individual-based model of malaria transmission dynamics. We also focused on predicting the isolated public health impact of an SMC candidate for a small range of deployment modalities and malaria-endemic settings. As such, this study does not provide precise predictions of the probable impact of SMC within a malaria control programme, but rather aims to identify guiding principles to inform clinical development and support candidate selection. Moreover, decisions made on the basis of evidence from modelling should be accompanied with a solid understanding of intervention dynamics from early clinical studies and clinical trial data, as well as evidence of feasible levels of coverage and adherence from implementation studies, as previously discussed.

In conclusion, this study provides evidence-based support for chemoprevention drug candidate design and selection for effectiveness of SMC programmes. Our results showed the importance of selecting and evaluating chemoprevention drugs for their duration of protection first and treatment efficacy second. Results also highlight the need to match SMC cycle timing to the protection duration of the drug, which would necessitate additional studies to assess the impact on acceptability, feasibility, and SMC coverage. Providing each eligible child with at least one chemoprevention cycle remains key, and drug candidates must be designed or selected for ease of administration, adherence, and community acceptance.

## Equitable partnership declaration

The authors of this paper have submitted an equitable partnership declaration (appendix 2). This statement allows researchers to describe how their work engages with researchers, communities, and environments in the countries of study. This statement is part of *The Lancet Global Health*'s broader goal to decolonise global health.

## Data sharing

All code and datasets are available on GitHub (https://github.com/lydiab-mayer/modelling-smc-drug-properties/tree/v1.0.0).

## Declaration of interests

We declare no competing interests.

## Supplementary Materials

Supplementary appendix 1

Equitable Partnership Declaration
