# Supplementary material for: Design and selection of drug properties to increase the public health impact of next-generation seasonal malaria chemoprevention: a modelling study
Source: Lancet Glob Health. 2024 Feb 14;12(3):e478–90. doi: 10.1016/S2214-109X(23)00550-8 (PMC10882206; doi:10.1016/S2214-109X(23)00550-8)
Supplement: Supplementary appendix 1 [file mmc1.pdf]

THE LANCET
Global Health

Supplementary appendix 1

This appendix formed part of the original submission and has been peer reviewed. We post it as supplied by the authors.

Supplement to: Braunack-Mayer L, Malinga J, Masserey T, et al. Design and selection of drug properties to increase the public health impact of next-generation seasonal malaria chemoprevention: a modelling study. *Lancet Glob Health* 2024; **12:** e478–90.

Contents

1. Methods 2
1.1. Mathematical transmission model 2
1.2. Scenarios and outcome measures 3
1.3. Intervention dynamics 4
1.4. Simulation and statistical analysis 6
2. Additional results 8
3. References 21

1. Methods

1.1. Mathematical transmission model

The potential public health impact of next-generation seasonal malaria chemoprevention (SMC) was predicted using an established and validated individual-based malaria transmission model, OpenMalaria (https://github.com/SwissTPH/openmalaria/wiki).[1] OpenMalaria has multiple model components, each of which captures a different set of malaria epidemiology and transmission assumptions and has been fully described previously.[2-4] Here we present an overview of the relevant dynamics for the model variant used in our study, which included an explicit mechanistic model of within-host parasite dynamics[5] and incorporated flexible pharmacokinetic/pharmacodynamic (PK/PD) model for drug action.[2]

OpenMalaria generates discrete, stochastic simulations of malaria infection and transmission from individual human hosts, tracked in five-day time steps. This model, which was fully described again in Reiker and colleagues,[3] with key components listed in table A1.1, and features: seasonally forced sub-models representing malaria parasite transmission within mosquitos; stochastic predictions of parasite densities within humans, determined by a malaria infection model and acquired immunity to asexual blood stage parasitaemia, and; the ability to track and distinguish between uncomplicated and severe episodes of clinical malaria, as well as direct and indirect malaria mortality. Together, these features enable detailed simulation of malaria public health outcomes for different interventions, such as the impact of malaria case management, drug-based chemoprevention interventions, and vector control.[6]

The within-host model variant adds a mechanistic feature of parasite dynamics within the human host, where each infection is modelled with a mechanistic *Plasmodium falciparum* parasite model adapted from Molineaux and colleagues,[5] and incorporates full PK/PD models of intervention dynamics. This model mechanistically describes the time-course of asexual *Plasmodium falciparum* parasitaemia following a single inoculation within an individual human host. This time-course is driven by a parasite growth rate and by the effect of three different immune responses to parasitaemia within the human host – innate and cross-variant immunity, acquired and variant specific immunity, and acquired and cross-variant immunity – each of which acts to reduce the parasite growth rate. By using a malaria transmission model with this realistic mathematical, within-host model of asexual parasitaemia, we capture a medical intervention's impact on both the time-course of asexual *P. falciparum* parasitaemia and changes to acquired immunity by modelling the drug's impact on parasite growth.

Name	Description and assumptions	References
**Key modelled epidemiological processes**		
Human malaria infection	• Determined by EIR, a model input that affects the force of infection in the simulated setting<br>• Human exposure to mosquitoes depends on age	1,7
Infection progression in humans: asexual parasite densities and immunity	• Several within-host models of asexual parasite densities are included in OpenMalaria. In each model, blood stage parasite density depends on the time since infection and is affected by naturally acquired immunity<br>1. Empirical model where expected densities of a single infection are sampled from a log-normal distribution and calibrated to malaria therapy data. The model captures between and within host variation and the duration of infection follows a log-normal distribution[8]<br>2. Explicit mechanistic within-host model of asexual densities, capturing innate, variant-specific, and variant-transcending acquired immunity[5]<br>• Immunity (both pre-erythrocytic and blood stage) develops progressively following consequent episodes of exposure to infection and decays exponentially<br>• Acquired immunity reduces parasite density of subsequent infections<br>• Multiple distinct infections are possible with cumulative parasite densities	1,2,5,7-9
Transmission from infected humans to mosquitoes	• Depends on the density of parasites present in the human, with gametocyte densities following between 10 and 20 days following asexual infection	1,4,10
Clinical illness, morbidity, mortality and anaemia	• Acute clinical illness depends on human host parasite densities and their pyrogenic threshold, which evolves over time depending on the individual exposure history<br>• Acute morbidity episodes can be uncomplicated or can evolve to severe episodes<br>• A proportion of the severe episodes leads to deaths<br>• Severe disease is also induced by age-related comorbidities, resulting in indirect severe disease and mortality	1,11-13
**Modelled characteristics of the transmission setting**		
Population age structure	• Flexible and informed by health and demographic surveillance data from Tanzania	13,14

Name	Description and assumptions	References
Sex and gender	• Model dynamics and outputs are not separated by sex or gender, due to the lack of appropriate data for model calibration to these characteristics	
Transmission seasonality	• Seasonally forced, the same transmission pattern is reproduced each year in absence of interventions	7,15
Case management	• Modelled through a comprehensive decision tree-based model, which determines treatment implications depending on the occurrence of clinical events such as fever and care seeking<br>• Its representation includes specification of diagnostic tests, effects of treatment, case fatality, case sequelae, and cure rates	16
Entomological setting	• Comprehensive simulation of the mosquito lifecycle and behavior towards human and animal hosts (biting, resting) embedded in a dynamic entomological model of the mosquito oviposition cycle<br>• Multiple vector species can be simulated simultaneously	17
**Modelled interventions**		
Vector control	• Available interventions are: long-lasting insecticide-treated nets (LLINs), indoor residual spraying (IRS), house screening, baited traps, repellents, and push-pull	17
Drugs and Vaccines	• Drugs and vaccines acting at various levels of the parasite life cycle (transmission blocking, anti-infective, blood stage clearance with PK/PD models)	2,18
Deployment characteristics	• In addition to case-management of clinical cases, interventions can be deployed in several ways:<br>1. Continuously to individuals by age, for example to infants for an expanded program of immunisation or for perennial malaria chemo-prevention<br>2. At specified times in a year or over multiple years, to targeted group of individuals (for example by age) for several cycles and specified coverages<br>• Interventions can be deployed by enrolling individuals into cohorts and tracking cohort outcomes, facilitating clinical trial simulation	a
**Simulation regimes and model variants**		
Time steps	• Simulation outputs are tracked every 1 or 5 days	
Model variants	• Varying assumptions in immunity decay, treatment, and heterogeneity of transmission result in 14 model variants	19
**Software availability and documentation**		

[a]Source code and wiki page available on GitHub: https://github.com/SwissTPH/openmalaria/

Table A1.1: **Summary of disease model characteristics, adapted from Golumbeanu et al. [20]Scenarios and outcome measures**

Term	Scenario
**Case management**	Results for two levels of health system access for first-line treatment of malaria were captured:<br>• A low probability of seeking care for clinical illness (10% within 14 days).<br>• A high probability of seeking care for clinical illness (50% within 14 days).<br>Treatment was assumed to clear all blood stage infections for one time-step.
**Primary outcomes**	We measured next-generation SMC's impact on three relative outcomes in the target population:<br>• **Clinical incidence reduction**: The percentage reduction in clinical incidence compared with a no intervention counterfactual, where incidence was defined as the number of new, uncomplicated malaria cases across the three-, four-, or five-month intervention period in the fifth year after deployment. Uncomplicated malaria was defined as an episode of symptomatic malaria, detectable by rapid diagnostic test with 94.2% specificity and a detection limit of 50 parasites per microlitre, where symptoms did not qualify as severe malaria.<br>• **Prevalence reduction**: The percentage reduction in prevalence compared with a no intervention counterfactual, where prevalence was defined as the proportion of all malaria infections (detectable by rapid diagnostic test or not) at the end of the three-, four-, or five-month intervention period in the fifth year after deployment.<br>• **Severe disease reduction**: The percent reduction in the number of severe malaria cases compared with a no intervention counterfactual, where severe cases of malaria were evaluated across the three-, four-, or five-month intervention period in the fifth year after deployment. An episode of severe malaria was an episode of symptomatic malaria, detectable by rapid diagnostic test with 94.2% specificity and a detection limit of 50 parasites per microlitre, with symptoms qualifying as severe malaria or with co-morbidities[1].<br>All outcomes were evaluated in the intervention's target population: children aged three to 59 or three to 119 months.
**Seasonality**	Differences in SMC's performance were captured across types of seasonal transmission by reporting results for archetypal seasonal profiles, where approximately 70% of cases occurred within three or five months in a given year (figure A1.2).
**SMC cycles**	The importance of covering the entire length of a region's highest risk period to malaria infection was evaluated by varying the number of cycles of SMC:

	• Three cycles of SMC, where the first cycle was deployed one month prior to peak malaria transmission, with additional cycles deployed in monthly intervals for the two subsequent cycles.<br>• Four cycles of SMC, where the first cycle was deployed one month prior to peak malaria transmission, with additional cycles deployed in monthly intervals for the three subsequent cycles.<br>• Five cycles of SMC, where the first cycle was deployed two months prior to peak malaria transmission, with additional cycles deployed in monthly intervals for the three subsequent months.
**Target population**	SMC was evaluated for two target populations:<br>• Children three to 59 months (5 years) old.<br>• Children three to 119 months (10 years) old.
**Transmission intensity**	SMC's performance was explored across a variety of malaria-endemic regions by providing results across a range of transmission intensities, from low (8%) to high (39%) annual baseline annual prevalence rate of *P. falciparum* patent infections (detectable by rapid diagnostic test with 94.2% specificity and a detection limit of 50 parasites per microlitre) in children aged two to ten years of age (*Pf*PR_{2-10}) compared with the no SMC intervention counterfactual.

Table A1.2: **Summary of simulation scenarios and outcome measures**

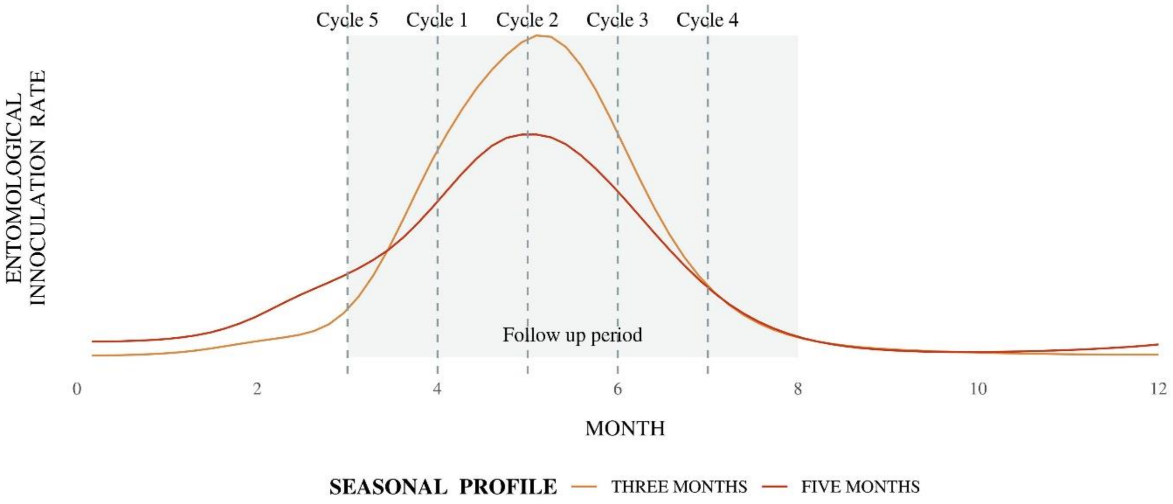

Figure A1.2: **Seasonality profiles and SMC cycle timing**

1.2. Intervention dynamics

Next-generation SMC with blood stage activity only

To model next-generation SMC drugs with blood stage activity only, we used a one-compartment pharmacokinetic/pharmacodynamic (PK/PD) model to describe a next-generation SMC drug's killing effect at time t as

$$E_{max} * \frac{C(t)^n}{C(t)^n + EC_{50}^n},$$

where $C(t)$ is the drug's concentration in mg/liter, E_{max} the maximum parasite killing rate per day, EC_{50} the concentration at which 50% of the maximum killing rate occurs in mg/liter, and n the slope of the drug's dose response curve. [21] The drug's concentration decays over time t with

$$C(t) = C_0 * e^{-\frac{ln(2)}{t_{1/2}}t},$$

Where C_0 is the drug's concentration at time zero and $t_{1/2}$ is the elimination half-life.

For simplicity, given that pharmacokinetic properties for next-generation SMC are still unknown, we assumed a piperaquine-like treatment schedule with a single dose administered daily for three days at a weight-based dosing schedule of 18 mg/kg.[22] Parameter values for the model's volume of distribution and EC_{50} were selected to represent piperaquine-like behavior,[23] as detailed in table 1.

We captured uncertainty around next-generation SMC's pharmacological properties by varying key parameters for drug efficacy and duration. To capture uncertainty about drug efficacy, we varied the E_{max} between two and 30. To represent uncertainty in a drug's duration of protection, we ranged the elimination half-life $t_{1/2}$ between five and 40 days. In this one-compartment PK/PD, uncertainty in drug duration could equally have been represented by ranging the EC_{50}. We varied the EC_{50} in our initial analyses and elected to present results for a fixed value. This modelling decision was made in order to limit the complexity of performing optimisation on two parameters for duration. The slope *n* was also varied in initial analyses but was found to have minimal contribution to model outcomes, which were presented for a slope of six.

The PK component of this model has only one compartment and assumes instantaneous absorption and, as such, may not reflect the complexity of a chemoprevention drug's exposure-response relationship. However, this simple approach to modelling SMC captured a next-generation drug's potential for activity against blood stage parasites without making complex assumptions about that drug's as-yet-unknown PK properties.

Next-generation SMC with dominant blood stage activity and initial, complete liver stage clearance

Some next-generation SMC candidates are likely to be partially active against liver stage parasites. For this reason, our 'dominant blood stage activity' model deployed the PK/PD model described above with the addition of one time-step of liver stage parasite clearance from the start of SMC administration.

Next-generation SMC with dominant liver stage activity and initial, complete blood stage clearance

To capture next-generation SMC's likely protective effect against liver stage parasites, we modelled the intervention's protective effect as a probability of preventing liver stage infection that decays over time, in combination with five days of blood and liver stage parasite clearance from the start of SMC administration. This approach, which was based on a previously calibrated model of SP-AQ,[24] has two key parameters: duration of protection, driven by the drug's elimination half-life and EC_{50}, and; initial probability of preventing infection, driven by the drug's maximal effect E_{max}. By modelling the intervention's protective effect over time, we could translate a drug's pharmacokinetics or pharmacodynamics to its expected public health impact. As for next-generation SMC with blood stage activity, we modelled a range of values for these key characteristics (table 1).

In-silico clinical trial modelling for the standard-of-care

We assessed each model's ability to accurately represent the dynamics of SP-AQ through in-silico clinical trial modelling, where we replicated SP-AQ's protective efficacy against clinical cases from a randomised non-inferiority trial of dihydroartemisinin-piperaquine (DHA-PPQ) to SP-AQ, conducted between 2009 and 2010 in Burkina Faso by Zongo and colleagues.[22] Adapting Burgert and colleagues' previous parameterization of SP-AQ to this same trial,[24] using the following approach:

1. Protective efficacy data for SP-AQ against clinical cases was extracted from figure 3 of Zongo and colleagues, and the trial was re-built in OpenMalaria using the settings described in table A1.3.
2. For each of the three mechanism-of-action models, we used latin-hypercube sampling to generate 500 samples of parameter values within the ranges described in table 1.
3. For each potential mechanism-of-action and for each parameter sample, we ran ten stochastic replicates of the in-silico clinical trial with OpenMalaria, simulating both the intervention arm of the trial and a no-intervention control arm.
4. Protective efficacy was calculated per time-step as
$$pe = 1 - \frac{cases\ per\ person_{Intervention\ arm}}{cases\ per\ person_{Control\ arm}}$$
where clinical cases were detectable by rapid diagnostic test.
5. Protective efficacy for each potential mechanism-of-action was considered to be sufficiently close to that of SP-AQ if the residual sum of squares (RSS) was within 0·1 standard deviations of the minimum RSS.

Parameter	Value	Reference
**Mosquito species**	*Anopheles funestus*: 42% indoor biting and 31% outdoor biting<br>*Anopheles gambiae*: 14% indoor biting and 13% outdoor biting	25

**Total entomological innoculation rate (EIR)**	350 for in-silico modelling of blood stage mechanism-of-actions<br>154 for in-silico modelling of liver stage mechanism-of-actions	..
**Malaria transmission seasonality (as monthly EIR)**	*Anopheles funestus*: 23 (Aug), 16 (Sep), 0 (Oct), 3.1 (Nov)<br>*Anopheles gambiae*: 40 (Aug), 50 (Sep), 16 (Oct), 6.4 (Nov)	25
**Access to treatment**	Effective coverage within 14-days of 50%, represented in OpenMalaria as 24.12% over 5-day time-steps	26,27
**Clinical malaria treatment pathway**	First-line treatment with arthemeter-lumefantrine<br>Second-line treatment with quinine	27
**Insecticide treated net coverage**	14% between 2006 and 2008, increased to 27% during the trial period	22,27
**SMC timing**	Cycles on 15-Aug, 15-Sep, and 15-Oct in 2009<br>Follow-up on 1-Sep, 1-Oct, and 1-Nov in 2009	22

Table A1.3: **OpenMalaria settings to perform an in-silico clinical trial with SP-AQ**

1.3. Simulation and statistical analysis

Following our previous methodology[20] and as shown in figure A1.3, our predictive target product profile framework began with simulation from the individual-based malaria transmission model over a discrete sample of input intervention parameters. 1000 samples were uniformly generated with Latin hypercube sampling and, for each sample, simulations were generated with 5 stochastic replicates. Following simulation, we used the *hetgp* package[28] to fit a heteroskedastic Gaussian Process regression model to emulate the relationship between input intervention parameters and their corresponding simulated outcomes. This step trained a computationally light model emulator of the individual-based malaria transmission model's complex, deterministic dynamics. Emulator performance was assessed using the R-squared correlation coefficient to evaluate correlation between true and predicted outcomes against a 10% hold-out set.

We then undertook a nonparametric variance-based sensitivity analysis of our model results to identify the extent to which a small change in an SMC drug's properties contributed to a change in its effectiveness. This was performed with the sensitivity package[29], using the Sobol-Jansen method[30] to compute Sobol total-order indices for two uniform samples of 50 000 input intervention parameters, generated with Latin hypercube sampling with 1000 bootstrap replicates. Sobol total-order indices represent the contribution of a given input variable to the variance of the output, called total-order or total-effect because this contribution includes any contribution to the variance by interactions with other input variables.

To identify desirable product characteristics for SMC, we linked a desired public health outcome with its required minimum intervention properties using an optimisation grid search. This was performed for blood stage models only by evaluating emulator predictions of public health outcomes on a sample of intervention properties (10 000 samples generated with Latin hypercube sampling) and identifying the estimated minimum property value whose 95% prediction interval was above the given target reduction in both clinical incidence and severe disease. This minimum was then aggregated by calculating the most conservative (maximum) value across outcomes (clinical incidence and severe disease reduction measured across the intervention period), SMC deployments (three, four, and five monthly cycles of SMC in a given year surrounding peak seasonality) and levels for other model parameters (elimination half-life and E_{max}).All analyses were conducted in R.[31]

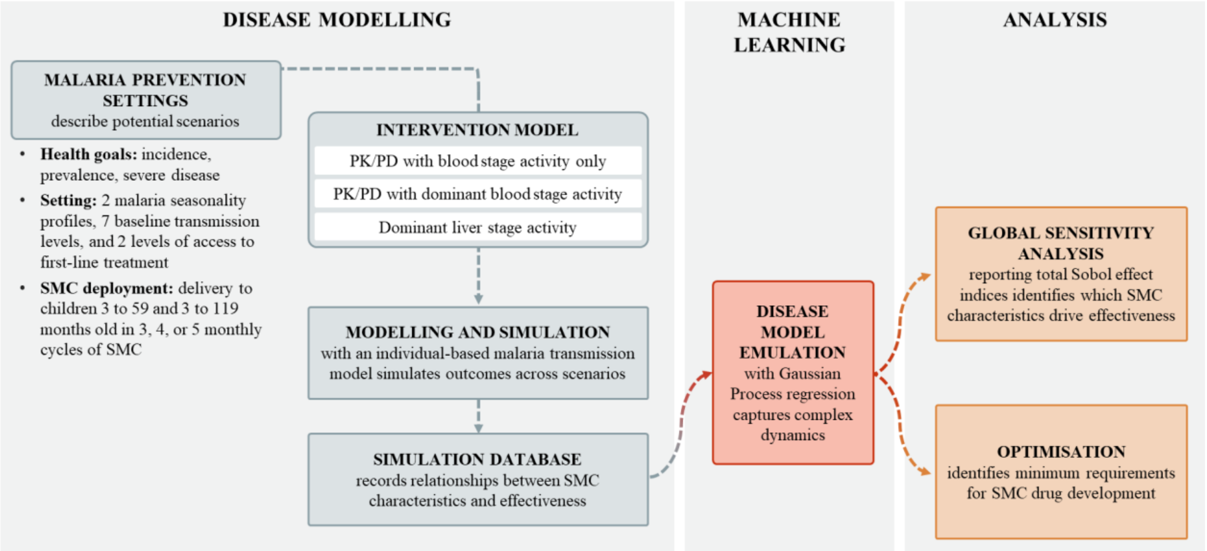

Figure A1.3: **Evidence generation framework combining individual-based malaria transmission modelling with statistical methods to support the identification of minimum necessary SMC product characteristics**

2. Additional results

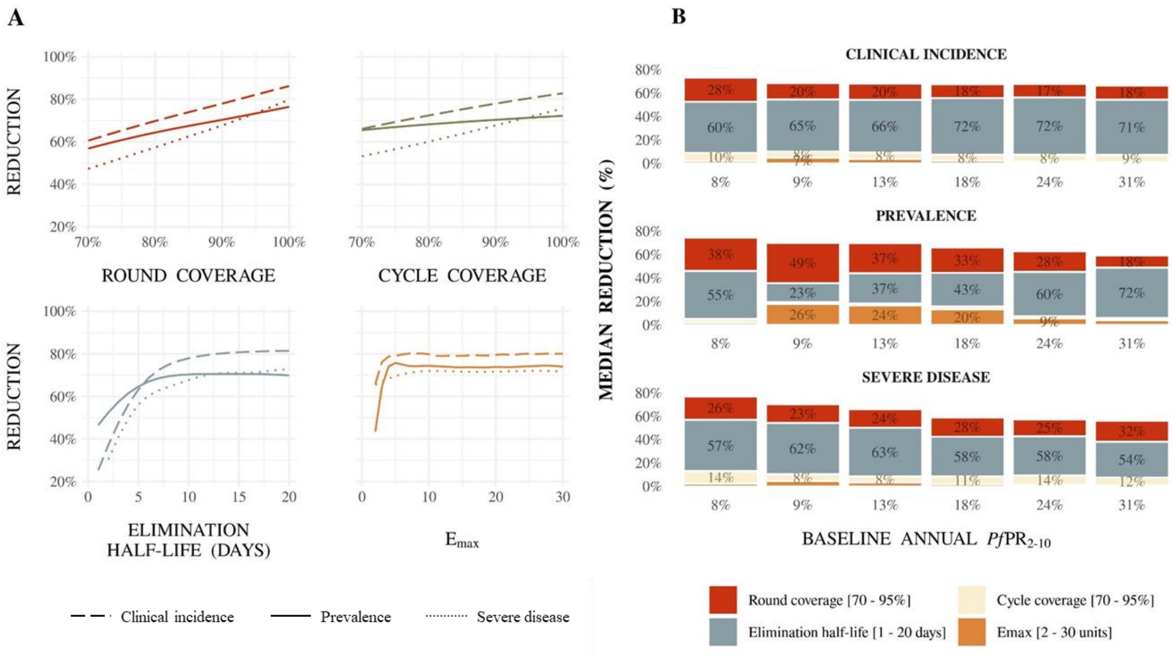

Figure A2.1: **For SMC with blood stage activity only, (A) illustrative Gaussian Process regression emulator predictions for the relationship between SMC properties and clinical incidence, prevalence, and severe disease reduction, and (B) drivers of impact on predicted clinical incidence, prevalence, and severe disease reduction for SMC in children three to 59 months of age, compared with a no intervention counterfactual**

(A) Results show 50% access to first-line treatment within 14 days and a five-month seasonal profile with baseline annual *Pf*$PR_{2\text{-}10}$ of 18% when SMC was deployed in four monthly cycles to children aged three to 59 months. Each panel shows the predicted reduction when all performance characteristics but the parameter of interest were held constant at: 90% round coverage, 90% cycle coverage, elimination half-life of 10 days, and a maximum parasite killing rate of 3.45 units.

(B) Bars indicate the total Sobol effect indices for key model parameters. Indices can be interpreted as the proportion of variation in the outcome attributable to a given change in each variable, along with its interactions with other variables. Bar heights indicate the median expected reduction across the modelled parameter range. Results are shown across prevalence settings (horizontal axis, baseline annual *Pf*$PR_{2\text{-}10}$) for an archetypal scenario of 50% access to first-line treatment within 14 days with a five-month seasonal profile, where SMC was deployed in four monthly intervals in a given year surrounding peak seasonality.

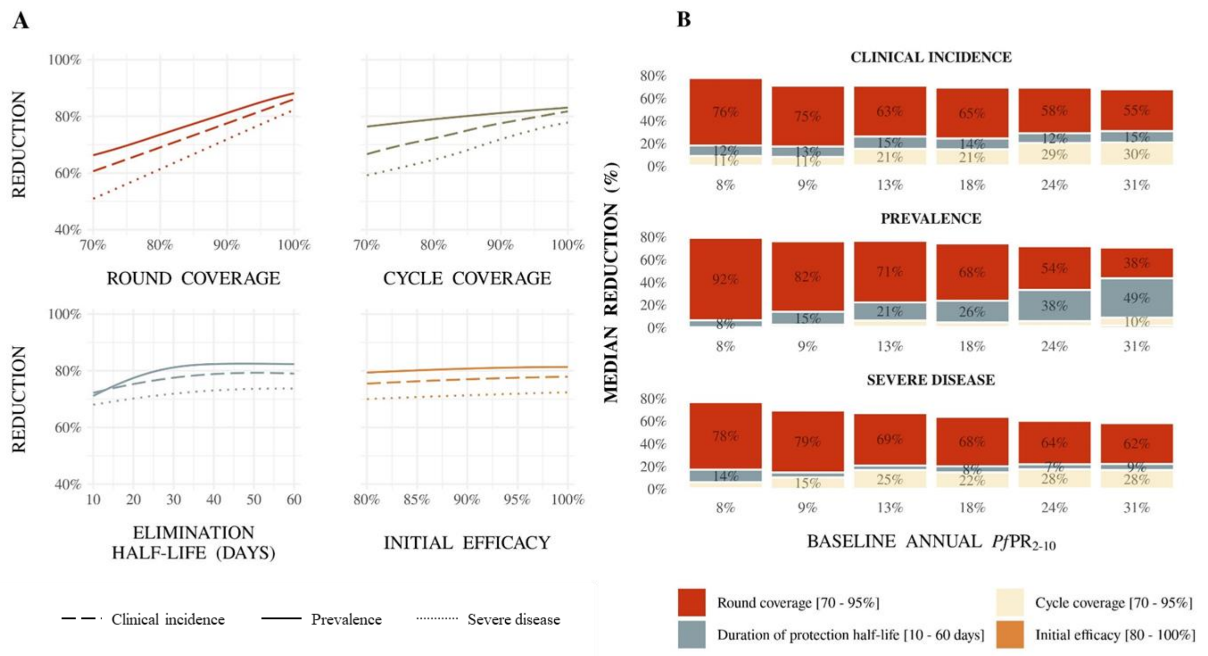

Figure A2.2: **For SMC with dominant liver stage activity and initial, complete blood stage clearance, (A) illustrative Gaussian Process regression emulator predictions for the relationship between SMC properties and clinical incidence, prevalence, and severe disease reduction, and (B) drivers of impact on predicted clinical incidence, prevalence, and severe disease reduction for SMC in children three to 59 months of age, compared with a no intervention counterfactual**

(A) Results show 50% access to first-line treatment within 14 days and a five-month seasonal profile with baseline annual *Pf*PR_{2-10} of 18% when SMC was deployed in four monthly cycles to children aged three to 59 months. Each panel shows the predicted reduction when all performance characteristics but the parameter of interest were held constant at: 90% round coverage, 90% cycle coverage, duration of protection half-life of 30 days, and initial efficacy of 95%.

(B) Bars indicate the total Sobol effect indices for key model parameters. Indices can be interpreted as the proportion of variation in the outcome attributable to a given change in each variable, along with its interactions with other variables. Bar heights indicate the median expected reduction across the modelled parameter range. Results are shown across prevalence settings (horizontal axis, baseline annual *Pf*PR_{2-10}) for an archetypal scenario of 50% access to first-line treatment within 14 days with a five-month seasonal profile, where SMC was deployed in four monthly intervals in a given year surrounding peak seasonality.

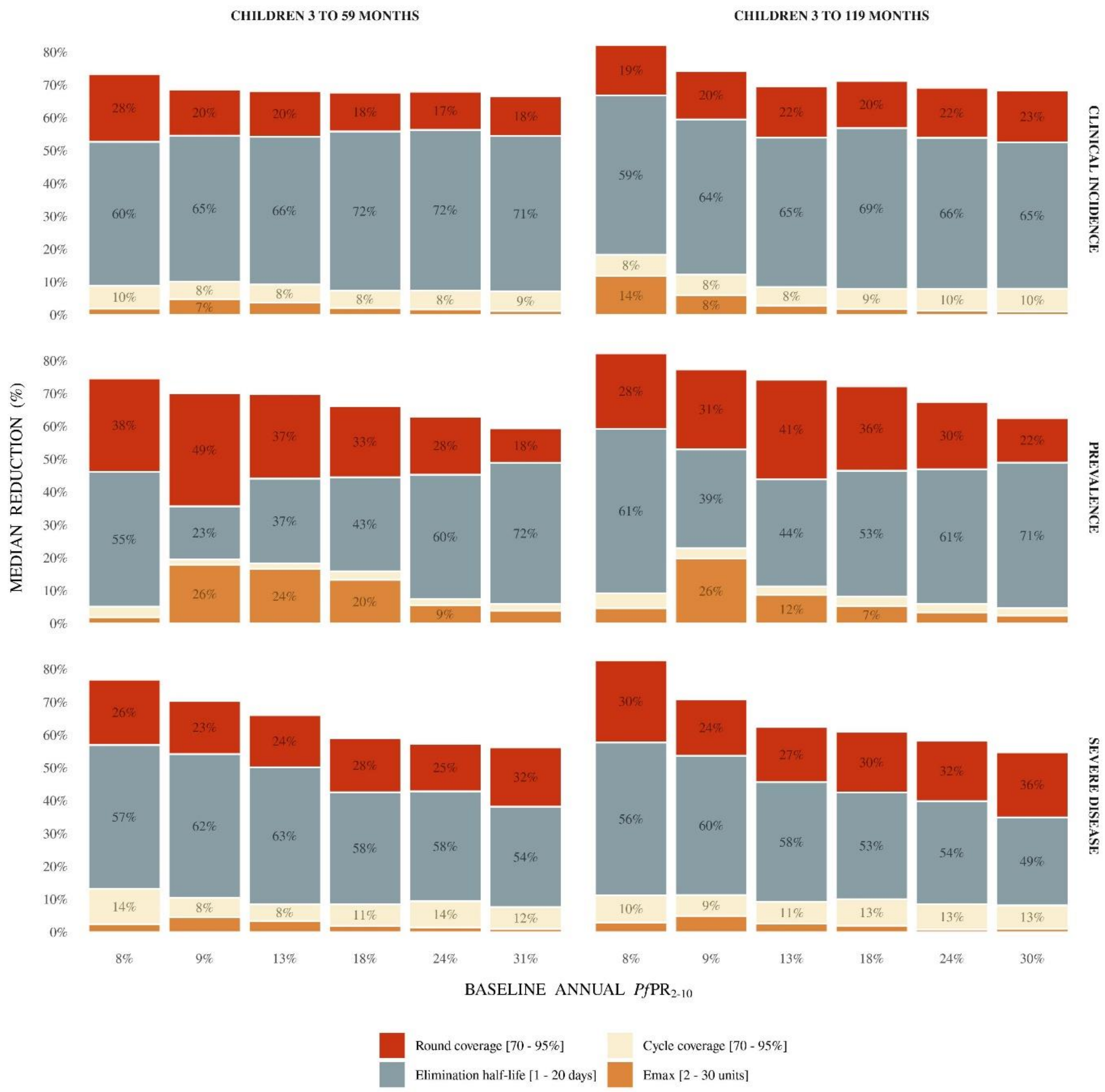

***Figure A2.3:* For SMC with blood stage activity only, drivers of impact on predicted levels of reduction of clinical incidence, prevalence, and severe disease in two scenarios – delivered to children aged three to 59 months or three to 119 months compared with the no intervention counterfactual**

Bars indicate the total Sobol effect indices for key model parameters. Indices can be interpreted as the proportion of variation in the outcome attributable to a given change in each variable, along with its interactions with other variables. Bar heights indicate the median expected reduction across the modelled parameter range. Results are shown across prevalence settings (horizontal axis, annual baseline *Pf*PR$_{2-10}$) for a scenario with 50% access to first-line treatment and a five-month seasonal profile where SMC was deployed in four monthly intervals in a given year.

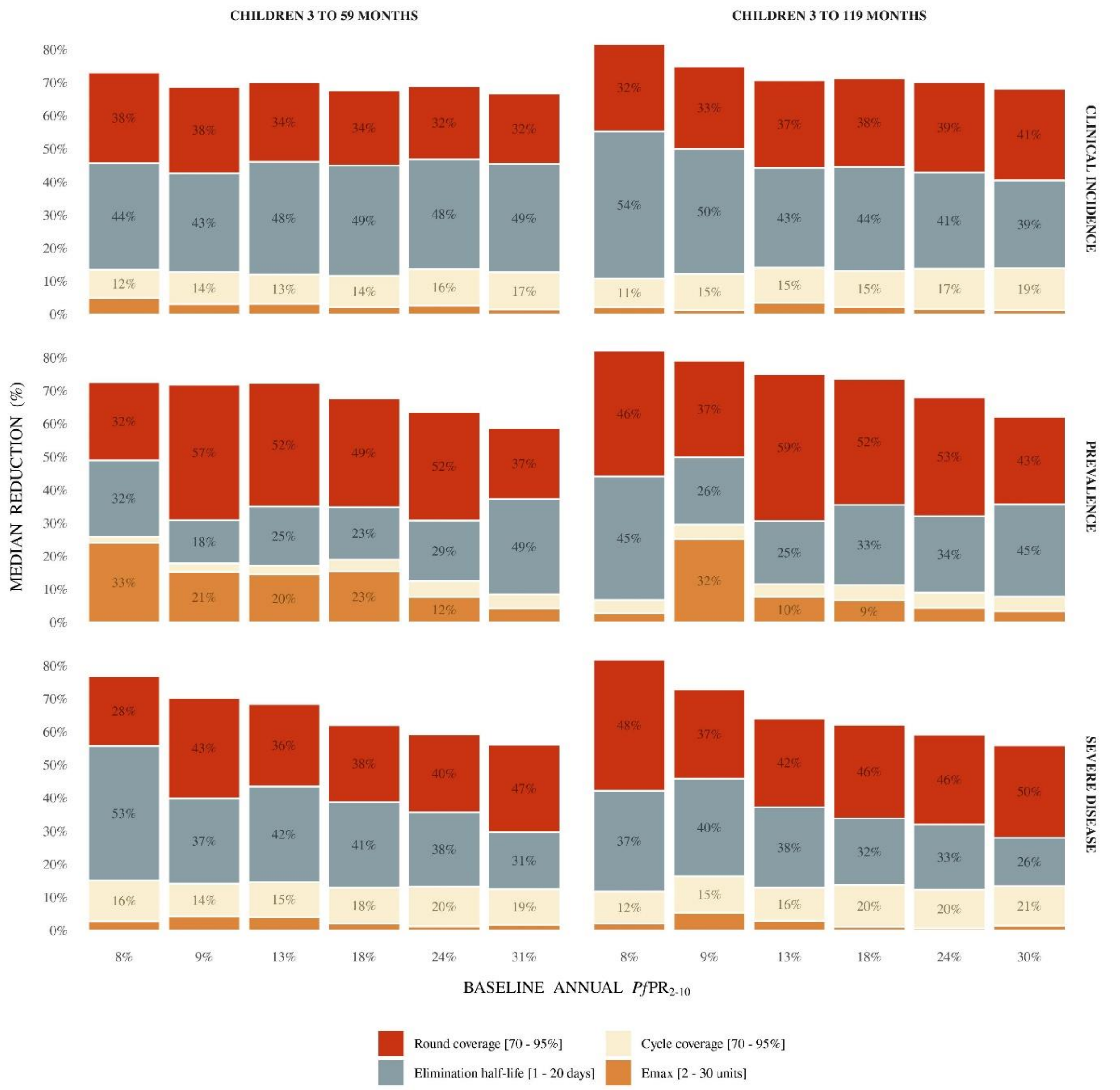

Figure A2.4: **For SMC with dominant blood stage activity and initial, complete liver stage clearance, drivers of impact on predicted levels of reduction of clinical incidence, prevalence, and severe disease in two scenarios – delivered to children aged three to 59 months or three to 119 months compared with the no intervention counterfactual**

Bars indicate the total Sobol effect indices for key model parameters. Indices can be interpreted as the proportion of variation in the outcome attributable to a given change in each variable, along with its interactions with other variables. Bar heights indicate the median expected reduction across the modelled parameter range. Results are shown across prevalence settings (horizontal axis, annual baseline *Pf*PR$_{2\text{-}10}$) for a scenario with 50% access to first-line treatment and a five-month seasonal profile where SMC was deployed in four monthly intervals in a given year.

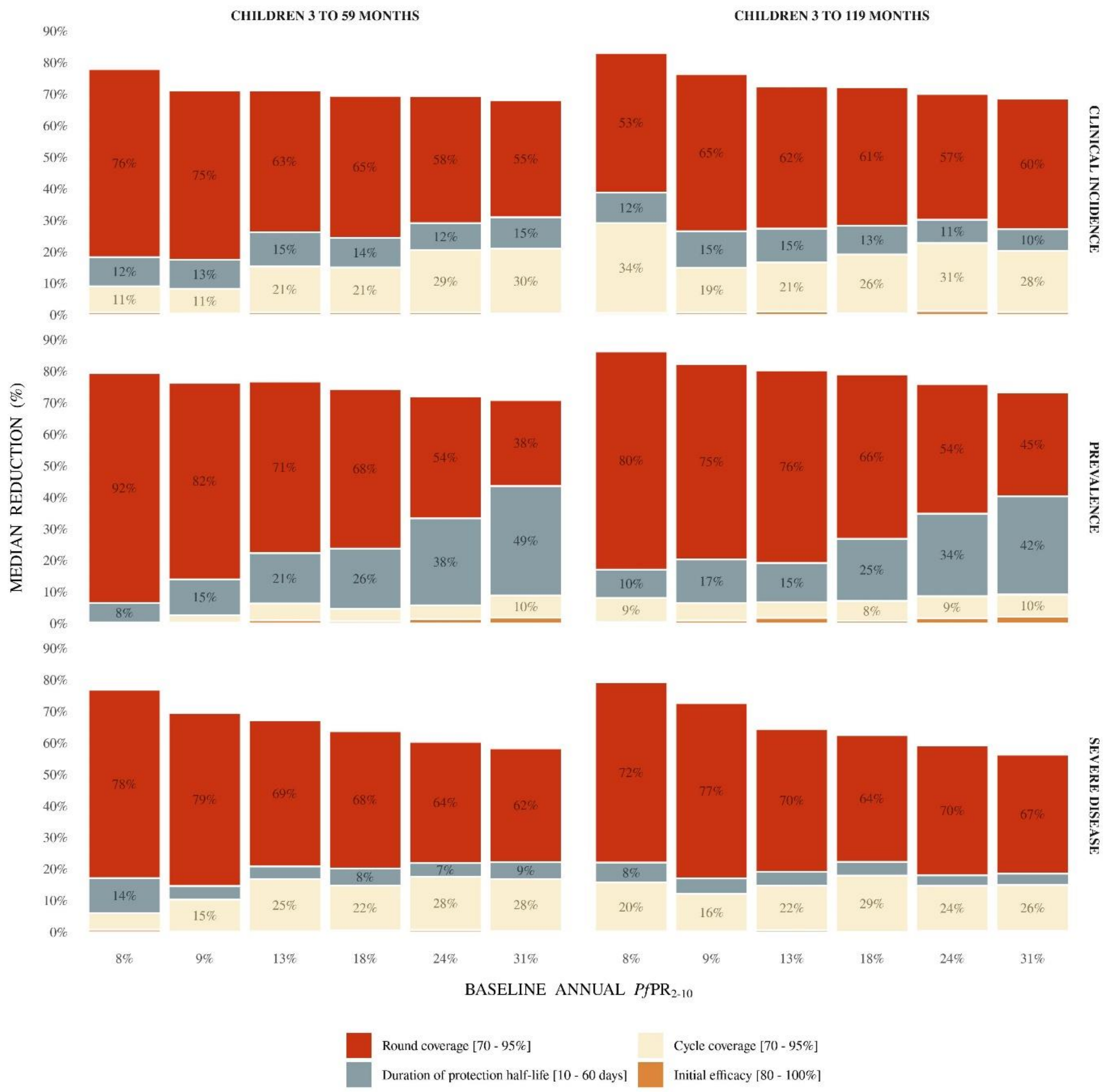

Figure A2.5: **For SMC with dominant liver stage activity and initial, complete blood stage clearance, drivers of impact on predicted levels of reduction of clinical incidence, prevalence, and severe disease in two scenarios – delivered to children aged three to 59 months or three to 119 months compared with the no intervention counterfactual**

Bars indicate the total Sobol effect indices for key model parameters. Indices can be interpreted as the proportion of variation in the outcome attributable to a given change in each variable, along with its interactions with other variables. Bar heights indicate the median expected reduction across the modelled parameter range. Results are shown across prevalence settings (horizontal axis, annual baseline *Pf*PR$_{2\text{-}10}$) for a scenario with 50% access to first-line treatment and a five-month seasonal profile where SMC was deployed in four monthly intervals in a given year.

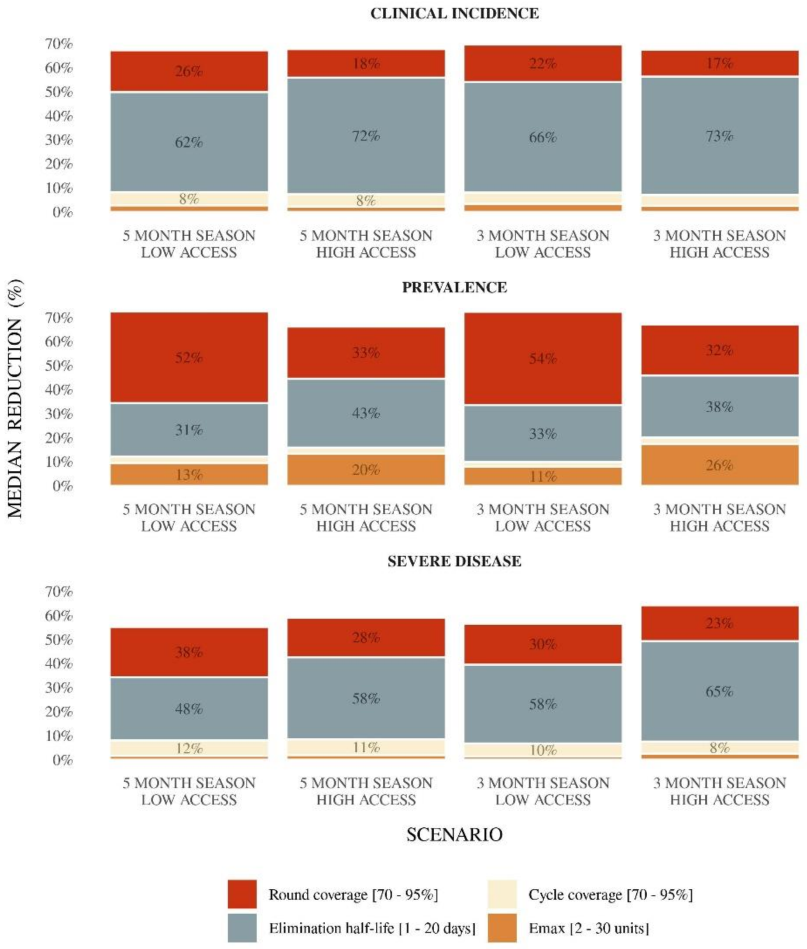

Figure A2.6: **For next-generation SMC with blood stage activity only, drivers of impact on predicted levels of reduction of clinical incidence, prevalence, and severe disease for SMC representing four scenarios – low (10%) and high (50%) levels of access to malaria treatment, and three- and five-month seasonal profiles – compared with the no intervention counterfactual**

Bars indicate the total Sobol effect indices for key model parameters. Indices can be interpreted as the proportion of variation in the outcome attributable to a given change in each variable, along with its interactions with other variables. Bar heights indicate the median expected reduction across the modelled parameter range. Results are shown for a scenario where SMC is deployed four times at monthly intervals in a given year in children aged three to 59 months, and where the annual baseline *Pf*PR$_{2-10}$ varied with seasonal profile and access from 14% to 24%.

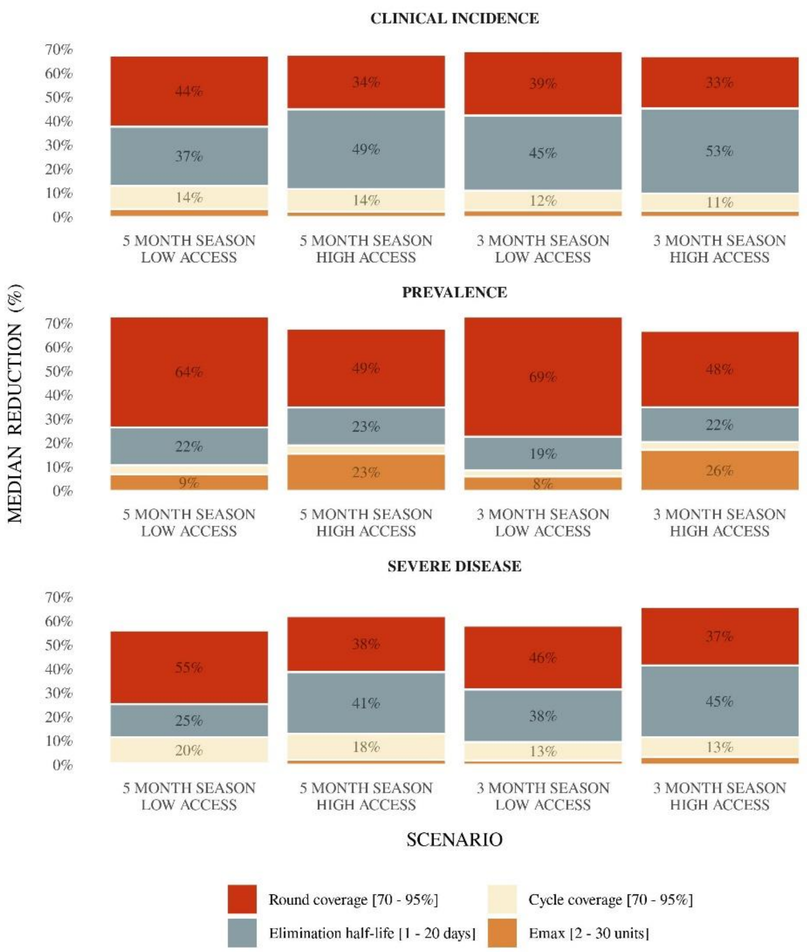

Figure A2.7: **For next-generation SMC with dominant blood stage activity and initial, complete liver stage clearance, drivers of impact on predicted levels of reduction of clinical incidence, prevalence, and severe disease for SMC representing four scenarios – low (10%) and high (50%) levels of access to malaria treatment, and three- and five-month seasonal profiles –compared with the no intervention counterfactual**

Bars indicate the total Sobol effect indices for key model parameters. Indices can be interpreted as the proportion of variation in the outcome attributable to a given change in each variable, along with its interactions with other variables. Bar heights indicate the median expected reduction across the modelled parameter range. Results are shown for a scenario where SMC is deployed four times at monthly intervals in a given year in children aged three to 59 months, and where the annual baseline *Pf*PR$_{2\text{-}10}$ varied with seasonal profile and access from 14% to 24%.

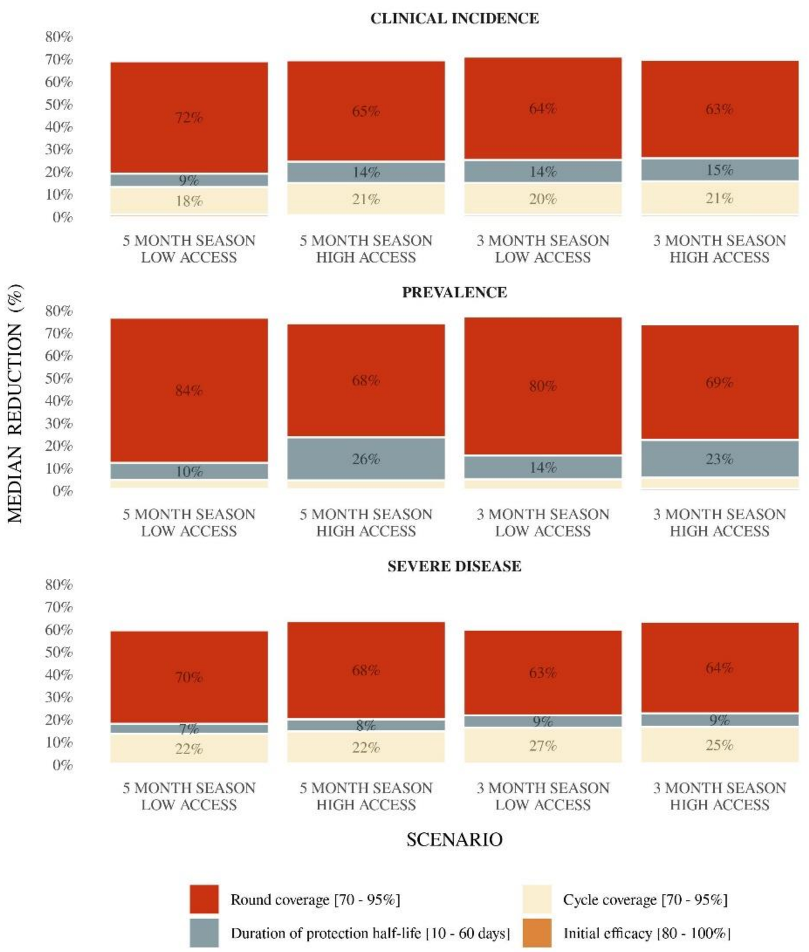

Figure A2.8: **For next-generation SMC with dominant liver stage activity and initial, complete blood stage clearance, drivers of impact on predicted levels of reduction of clinical incidence, prevalence, and severe disease for SMC representing four scenarios – low (10%) and high (50%) levels of access to malaria treatment, and three- and five-month seasonal profiles –compared with the no intervention counterfactual**

Bars indicate the total Sobol effect indices for key model parameters. Indices can be interpreted as the proportion of variation in the outcome attributable to a given change in each variable, along with its interactions with other variables. Bar heights indicate the median expected reduction across the modelled parameter range. Results are shown for a scenario where SMC is deployed four times at monthly intervals in a given year in children aged three to 59 months, and where the annual baseline *Pf*PR$_{2\text{-}10}$ varied with seasonal profile and access from 14% to 24%.

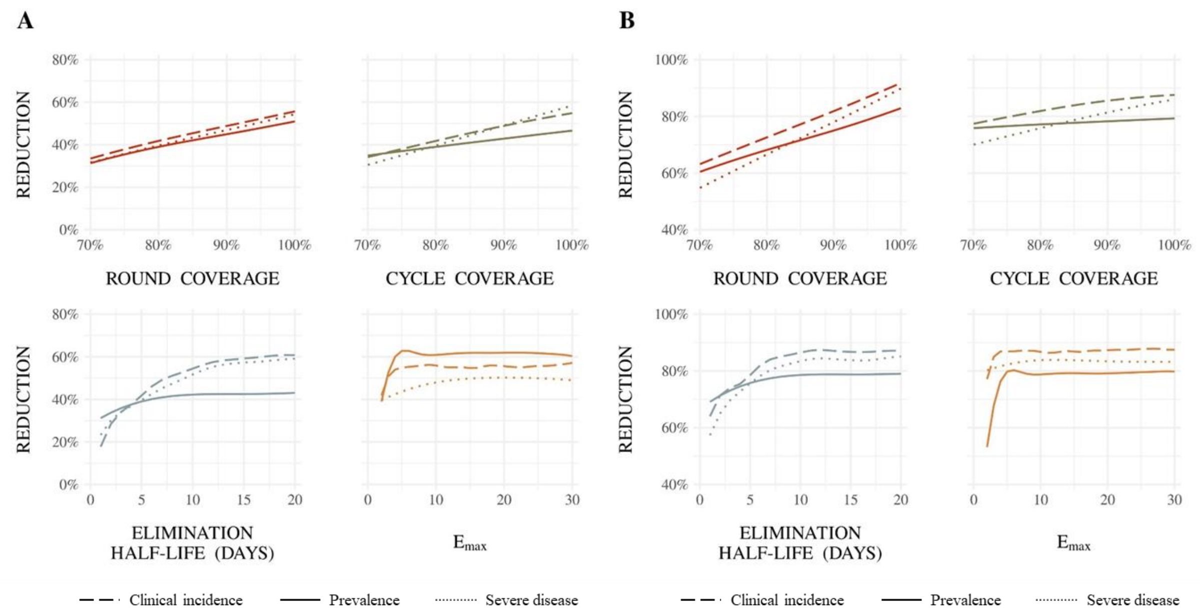

Figure A2.9: **For SMC with dominant blood stage activity and initial, complete liver stage clearance, (A) illustrative Gaussian Process regression emulator predictions for the relationship between SMC properties and their direct effects on clinical incidence, prevalence, and severe disease reduction for a drug profile with low coverage and low protective efficacy, and (B) illustrative Gaussian Process regression emulator predictions for the relationship between SMC properties and their indirect effects on clinical incidence, prevalence, and severe disease reduction for a drug profile with high coverage and high protective efficacy**

(A) Results show 50% access to first-line treatment within 14 days and a five-month seasonal profile with baseline annual *Pf*PR$_{2-10}$ of 18% when SMC was deployed in four monthly cycles to children aged three to 59 months. Each panel shows the predicted reduction when all performance characteristics but the parameter of interest were held constant at: 80% round coverage, 80% cycle coverage, an elimination half-life of 5 days, and a maximum parasite killing rate of 2 units.

(B) Results show 50% access to first-line treatment within 14 days and a five-month seasonal profile with baseline annual *Pf*PR$_{2-10}$ of 18% when SMC was deployed in four monthly cycles to children aged three to 59 months. Each panel shows the predicted reduction – evaluated in SMC naïve children aged 60 to 119 months in the first intervention year – when all performance characteristics but the parameter of interest were held constant at: 95% round coverage, 95% cycle coverage, an elimination half-life of 15 days, and a maximum parasite killing rate of 10 units.

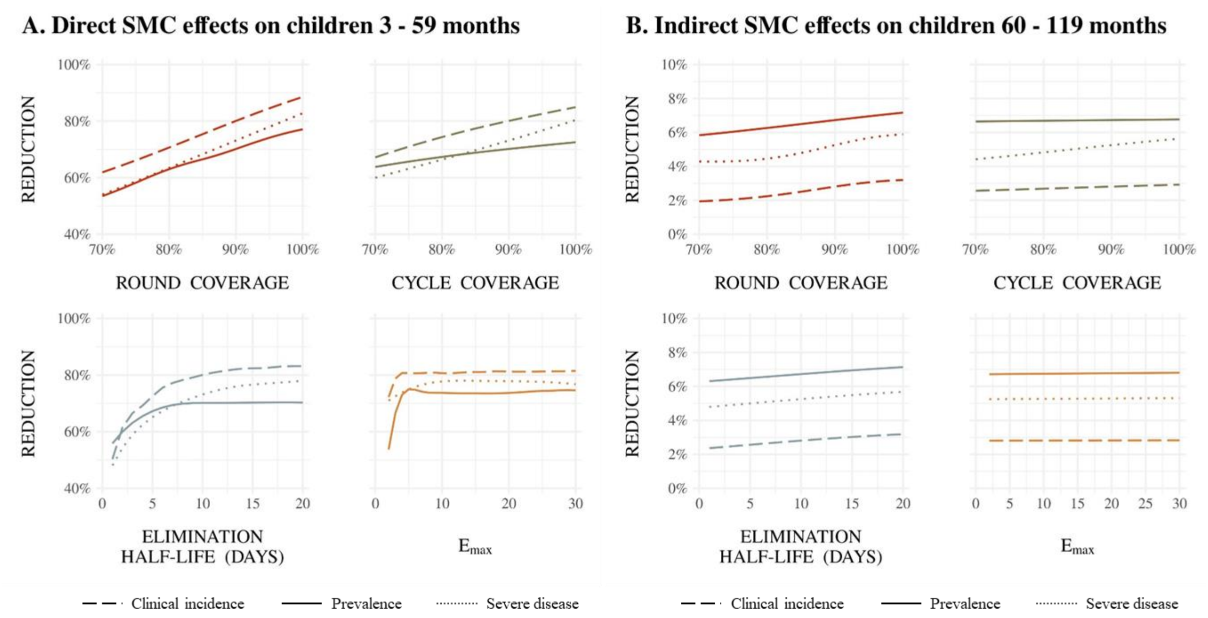

Figure A2.10: **For SMC with dominant blood stage activity and initial, complete liver stage clearance, (A) illustrative Gaussian Process regression emulator predictions for the relationship between SMC properties and their direct effects on clinical incidence, prevalence, and severe disease reduction in children aged three to 59 months receiving SMC, and (B) illustrative Gaussian Process regression emulator predictions for the relationship between SMC properties and their indirect effects on clinical incidence, prevalence, and severe disease reduction in children aged 60 to 119 months not receiving SMC**

(A) Results show 50% access to first-line treatment within 14 days and a five-month seasonal profile with baseline annual *Pf*PR$_{2-10}$ of 18% when SMC was deployed in four monthly cycles to children aged three to 59 months. Each panel shows the predicted reduction – evaluated in the first intervention year – when all performance characteristics but the parameter of interest were held constant at: 90% round coverage, 90% cycle coverage, an elimination half-life of 10 days, and a maximum parasite killing rate of 3.45 units.

(B) Results show 50% access to first-line treatment within 14 days and a five-month seasonal profile with baseline annual *Pf*PR$_{2-10}$ of 18% when SMC was deployed in four monthly cycles to children aged three to 59 months. Each panel shows the predicted reduction – evaluated in SMC naïve children aged 60 to 119 months in the first intervention year – when all performance characteristics but the parameter of interest were held constant at: 90% round coverage, 90% cycle coverage, an elimination half-life of 10 days, and a maximum parasite killing rate of 3.45 units.

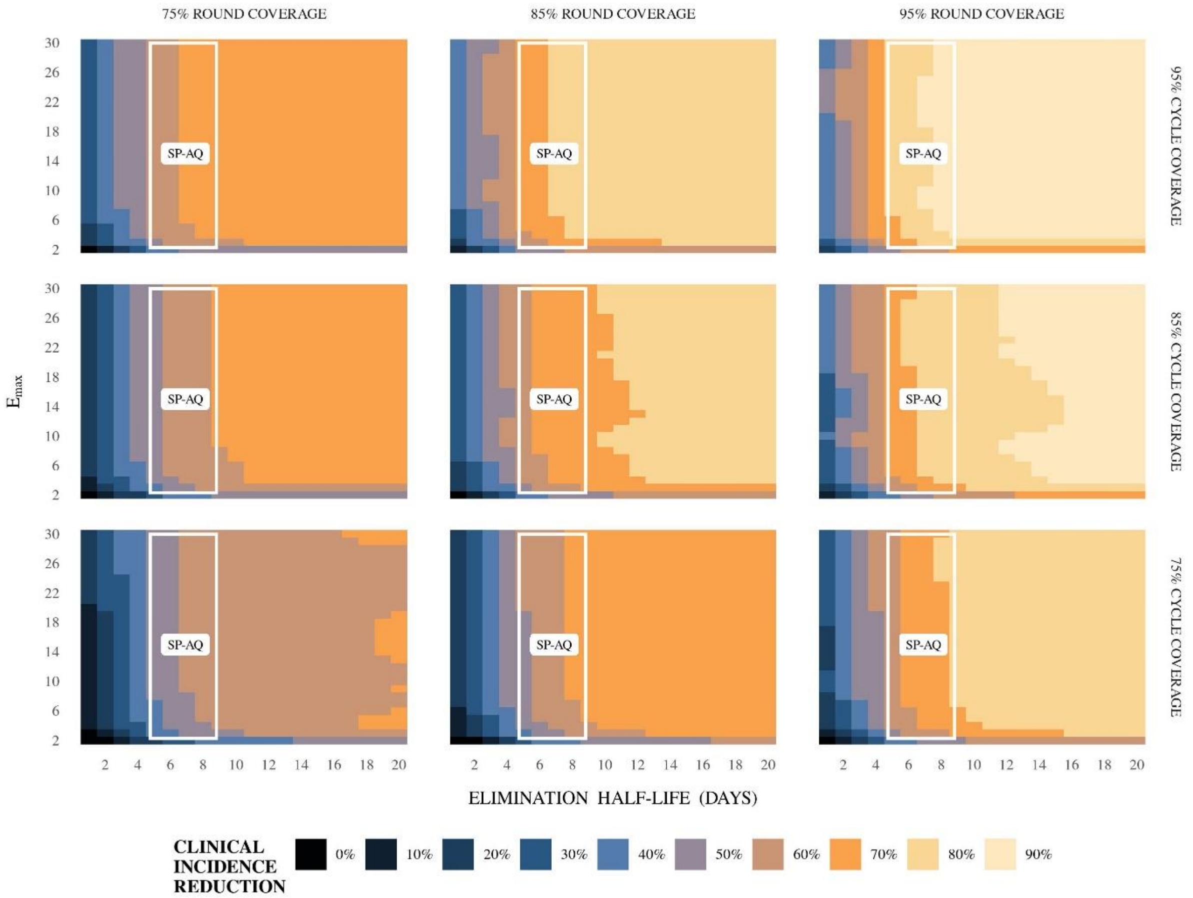

Figure A2.11: **For SMC with blood stage activity only, impact of a change in coverage on the predicted relative reduction in clinical incidence (measured over a four-month intervention period) following SMC compared with a no intervention counterfactual**

Each square in the grid indicates the predicted reduction (rounded to the nearest 10%) if an intervention with the given elimination half-life (horizontal axis) and maximum parasite killing rate (vertical axis) were deployed, assuming a slope of six. Variation in this figure is driven by the combined impact of stochastic uncertainty and emulator prediction error. Results are shown for children aged three and 59 months for a five-month seasonal profile with an baseline annual *Pf*PR$_{2\text{-}10}$ of 18%, where access to first-line treatment was 50% within 14 days and where SMC was deployed four times at monthly intervals in a given year surrounding peak seasonality. Each panel represents results for a different level of SMC round coverage (75%, 85%, and 95%) and cycle coverage (75%, 85%, and 95%). The white region indicates the space of parameter profiles whose RSS (in-silico protective efficacy calculated in comparison to SP-AQ's protective efficacy) falls within 0.1 standard deviations of the minimum (see figure 1).

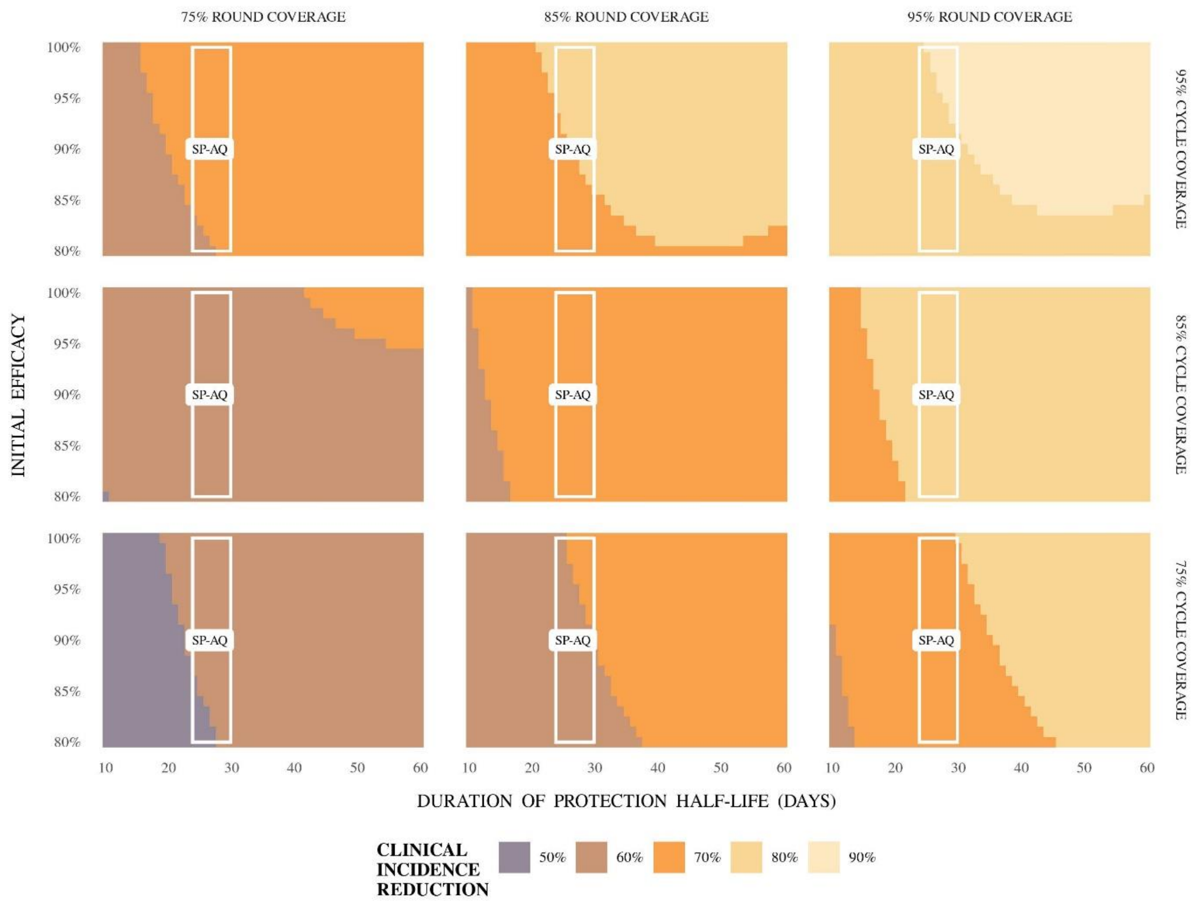

Figure A2.12: **For SMC with dominant liver stage activity and initial, complete blood stage clearance, impact of a change in coverage on the predicted relative reduction in clinical incidence (measured over a four-month intervention period) following SMC compared with a no intervention counterfactual**

Each square in the grid indicates the predicted reduction (rounded to the nearest 10%) if an intervention with the given duration of protection half-life (horizontal axis) and initial efficacy (vertical axis) were deployed. Variation in this figure is driven by the combination of combined impact of stochastic uncertainty and emulator prediction error. Results are shown for children aged three and 59 months for a five-month seasonal profile with an baseline annual *Pf*PR$_{2\text{-}10}$ of 18%, where access to first-line treatment was 50% within 14 days and where SMC was deployed four times at monthly intervals in a given year surrounding peak seasonality. Each panel represents results for a different level of SMC round coverage (75%, 85%, and 95%) and cycle coverage (75%, 85%, and 95%). The white region indicates the space of parameter profiles whose RSS (in-silico protective efficacy calculated in comparison to SP-AQ's protective efficacy) falls within 0.1 standard deviations of the minimum (see figure 1).

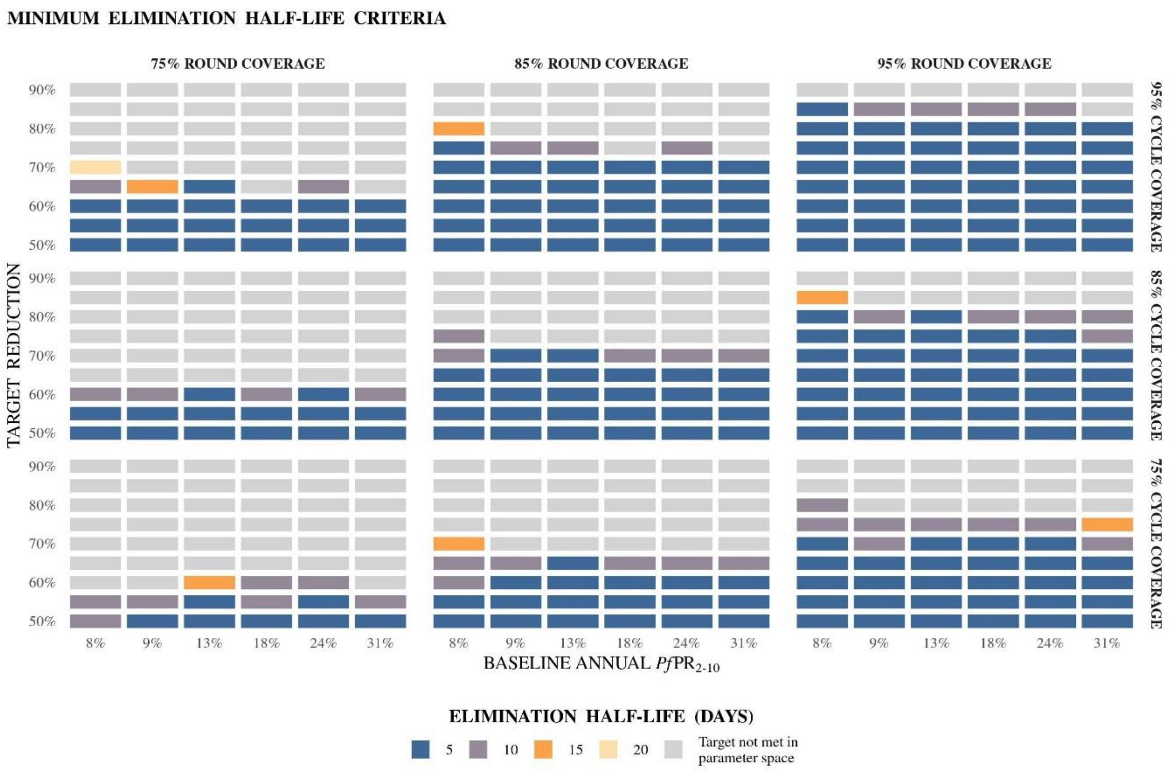

Figure A2.13: **Predicted minimum elimination half-life values for SMC with dominant blood stage activity and initial, complete liver stage clearance to achieve target reductions in clinical incidence and severe disease**

Summary of the predicted minimum elimination half-life criteria for SMC with dominant blood stage activity and initial, complete liver stage clearance towards achieving a target clinical incidence reduction for varying levels of SMC round and cycle coverage, shown for a five-month seasonal profile in a setting with 50% access to first-line treatment, where SMC was deployed in four monthly cycles to children three to 59 months old. Results show the estimated minimum elimination half-life with a 95% prediction interval above the given target reduction in clinical incidence (vertical axis), compared with a no intervention counterfactual and measured across the four-month intervention period. Minimum criteria were calculated across E_{max} levels.

3. References

1 Smith T, Killeen GF, Maire N, et al. Mathematical modeling of the impact of malaria vaccines on the clinical epidemiology and natural history of Plasmodium falciparum malaria: Overview. *Am J Trop Med Hyg* 2006; **75**: 1–10.

2 Masserey T, Lee T, Golumbeanu M, et al. The influence of biological, epidemiological, and treatment factors on the establishment and spread of drug-resistant Plasmodium falciparum. *Elife* 2022; **11**: e77634.

3 Reiker T, Golumbeanu M, Shattock A, et al. Emulator-based Bayesian optimization for efficient multi-objective calibration of an individual-based model of malaria. *Nat Commun* 2021; **12**: 7212.

4 Ross A, Killeen G, Smith T. Relationships between host infectivity to mosquitoes and asexual parasite density in Plasmodium falciparum. *Am J Trop Med Hyg* 2006; **75**: 32–7.

5 Molineaux L, Diebner HH, Eichner M, Collins WE, Jeffery GM, Dietz K. Plasmodium falciparum parasitaemia described by a new mathematical model. *Parasitology* 2001; **122**: 379–91.

6 Smith T, Maire N, Ross A, et al. Towards a comprehensive simulation model of malaria epidemiology and control. *Parasitology* 2008; **135**: 1507–16.

7 Smith T, Maire N, Dietz K, et al. Relationship between the entomologic inoculation rate and the force of infection for Plasmodium falciparum malaria. *Am J Trop Med Hyg* 2006; **75**: 11–8.

8 Maire N, Smith T, Ross A, Owusu-Agyei S, Dietz K, Molineaux L. A model for natural immunity to asexual blood stages of Plasmodium falciparum malaria in endemic areas. *Am J Trop Med Hyg* 2006; **75**: 19–31.

9 Collins WE, Jeffery GM. A retrospective examination of the patterns of recrudescence in patients infected with Plasmodium falciparum. *Am J Trop Med Hyg* 1999; **61**: 44–8.

10 Killeen GF, Ross A, Smith T. Infectiousness of malaria-endemic human populations to vectors. *Am J Trop Med Hyg* 2006; **75**: 38–45.

11 Ross A, Maire N, Molineaux L, Smith T. An epidemiologic model of severe morbidity and mortality caused by Plasmodium falciparum. *Am J Trop Med Hyg* 2006; **75**: 63–73.

12 Ross A, Smith T. The effect of malaria transmission intensity on neonatal mortality in endemic areas. *Am J Trop Med Hyg* 2006; **75**: 74–81.

13 Smith T, Ross A, Maire N, Rogier C, Trape JF, Molineaux L. An epidemiologic model of the incidence of acute illness in Plasmodium falciparum malaria. *Am J Trop Med Hyg* 2006; **75**: 56–62.

14 Ekstrom AM, Clark J, Byass P, et al. INDEPTH Network: contributing to the data revolution. *Lancet Diabetes Endocrinol* 2016; **4**: 97.

15 Stuckey EM, Smith T, Chitnis N. Seasonally dependent relationships between indicators of malaria transmission and disease provided by mathematical model simulations. *PLoS Comput Biol* 2014; **10**: e1003812.

16 Tediosi F, Maire N, Smith T, et al. An approach to model the costs and effects of case management of Plasmodium falciparum malaria in sub-saharan Africa. *Am J Trop Med Hyg* 2006; **75**: 90–103.

17 Chitnis N, Hardy D, Smith T. A periodically-forced mathematical model for the seasonal dynamics of malaria in mosquitoes. *Bull Math Biol* 2012; **74**: 1098–124.

18 Penny MA, Maire N, Studer A, Schapira A, Smith TA. What should vaccine developers ask? Simulation of the effectiveness of malaria vaccines. *PLoS One* 2008; **3**: e3193.

19 Smith T, Ross A, Maire N, et al. Ensemble modeling of the likely public health impact of a pre-erythrocytic malaria vaccine. *PLoS Med* 2012; **9**: e1001157.

20 Golumbeanu M, Yang GJ, Camponovo F, et al. Leveraging mathematical models of disease dynamics and machine learning to improve development of novel malaria interventions. *Infect Dis Poverty* 2022; **11**.

21 Winter K, Hastings IM. Development, evaluation, and application of an in silico model for antimalarial drug treatment and failure. *Antimicrob Agents Chemother* 2011; **55**: 3380–92.

22 Zongo I, Milligan P, Compaore YD, et al. Randomized Noninferiority Trial of Dihydroartemisinin-Piperaquine Compared with Sulfadoxine-Pyrimethamine plus Amodiaquine for Seasonal Malaria Chemoprevention in Burkina Faso. *Antimicrob Agents Chemother* 2015; **59**: 4387–96.

23 Staehli Hodel EM, Guidi M, Zanolari B, et al. Population pharmacokinetics of mefloquine, piperaquine and artemether-lumefantrine in Cambodian and Tanzanian malaria patients. *Malar J* 2013; **12**: 235.

24 Burgert L, Reiker T, Golumbeanu M, Möhrle JJ, Penny MA. Model-informed target product profiles of long-acting-injectables for use as seasonal malaria prevention. *PLOS Glob* 2022; **2**: e0000211.

25 Dabire KR, Baldet T, Diabate A, et al. Anopheles funestus (Diptera: Culicidae) in a humid savannah area of western Burkina Faso: bionomics, insecticide resistance status, and role in malaria transmission. *J Med Entomol* 2007; **44**: 990–7.

26 Penny MA, Maire N, Bever CA, et al. Distribution of malaria exposure in endemic countries in Africa considering country levels of effective treatment. *Malar J* 2015; **14**: 384.
27 World Health Organization. World Malaria Report 2008. Geneva, 2008 (https://www.who.int/publications/i/item/9789241563697).
28 Binois M, Gramacy RB. hetGP: Heteroskedastic Gaussian Process Modeling and Sequential Design in R. *J Stat Softw* 2021; **98**: 1–44.
29 Iooss B, Da Veiga S, Janon A, Pujol G. Global sensitivity analysis of model outputs. R; 2021.
30 Sobol IM. Global sensitivity indices for nonlinear mathematical models and their Monte Carlo estimates. *Math Comput Sim* 2001; **55**: 271–80.
31 R Core Team. R: A Language and Environment for Statistical Computing. Vienna, Austria: R Foundation for Statistical Computing; 2019.
