# Supplementary material for: Design and selection of drug properties to increase the public health impact of next-generation seasonal malaria chemoprevention: a modelling study
Source: Lancet Glob Health. 2024 Feb 14;12(3):e478–90. doi: 10.1016/S2214-109X(23)00550-8 (PMC10882206; doi:10.1016/S2214-109X(23)00550-8)
Supplement: Equitable Partnership Declaration [file mmc2.pdf]

THE LANCET
Global Health

Supplementary appendix 2

This Equitable Partnership Declaration (EPD) was submitted by the authors, and we reproduce it as supplied. It has not been peer reviewed. *The Lancet*'s editorial processes have not been applied to the EPD.

Supplement to: Braunack-Mayer L, Malinga J, Masserey T, et al. Design and selection of drug properties to increase the public health impact of next-generation seasonal malaria chemoprevention: a modelling study. *Lancet Glob Health* 2024; **12:** e478–90.

Equitable Partnership Declaration questions

This Equitable Partnership Declaration is a statement being published online alongside papers at *The Lancet Global Health*, as a separate appendix, to allow researchers to describe how their work engages with researchers, communities, and environments in the countries of study. This is part of our broader goal to decolonise global health, handing control and leadership of research to academics and clinicians who are based in the regions of study, and to affected communities.

Please answer all questions with as much detail as possible, noting that all included information will be published open-access and it will be freely available online to all who wish to read it. If a question does not apply to your study, please state "Not applicable".

The format of and questions in this statement are currently in a pilot phase. Please email Dr Liam Messin (Liam.Messin@lancet.com; deputy editor) and Dr Kate McIntosh (Kate.McIntosh@lancet.com; senior editor) with any feedback, particularly if you find any questions unclear.

Researcher considerations

1. Please detail the involvement that researchers who are based in the region(s) of study had during a) study design; b) clinical study processes, such as processing blood samples, prescribing medication, or patient recruitment; c) data interpretation; and d) manuscript preparation, commenting on all aspects. If they were not involved in any of these aspects, please explain why.

This question is intended for international partnerships; if all your authors are based in the area of study, this question is not applicable.

This should include a thorough description of their leadership role(s) in the study. Are local researchers named in the author list or the acknowledgements, or are they not mentioned at all (and, if not, why)? Please also describe the involvement of early career researchers based in the location of the study. Some of this information might be repeated from the Contributors section in the manuscript. Note: we adhere to ICMJE authorship criteria when deciding who should be named on a paper.

**a) Study design:**<br>Our mathematical modelling study was designed with collaborators from several countries and all analyses were conducted in Basel, Switzerland. Although our study was based in Switzerland, we made every effort to ensure that the international scope of this work was represented by the diversity of our research team and the scope of our engagement with the malaria medical product development community. Research questions and methods were designed in partnership with both early career and late career researchers who have lived experience in malaria-endemic countries (both as origin). These researchers are named in the author list. In addition, our research questions were informed by consultations with chemoprevention drug and guideline developers as part of the 2021 convening 'Malaria Prevention: Shaping Next-Gen Medical Interventions'. These consultations included individuals from across the malaria product development lifecycle and from both the Global North and Global South. The participants of this convening are acknowledged in our Acknowledgements section and two participants are named in the author list.
**b) Clinical study processes:**<br>No clinical data was collected as part of our study.

**c) Data interpretation:**
As above, data were interpreted by a diverse team of both early career and late career researchers, including researchers who have lived experience in malaria-endemic countries. These researchers are named in the author list.
**d) Manuscript preparation:**
As above, the manuscript was prepared by a diverse team of both early career and late career researchers, including researchers who have lived experience in malaria-endemic countries. These researchers are named in the author list.

2. Were the data used in your study collected by authors named on the paper, or have they been extracted from a source such as a national survey? ie, is this a secondary analysis of data that were not collected by the authors of this paper. If the authors of this paper were not involved in data collection, how were data interpreted with sufficient contextual knowledge?

   The Lancet Global Health *believe contextual understanding is crucial for informed data analysis and interpretation.*

The data used in our study was simulated from a mathematical model using computational resources based in Basel, Switzerland, on the University of Basel's Center for Scientific Computing (SciCore). This data was simulated and analysed by LBM and interpreted together with other named authors.

3. How was funding used to remunerate and enhance the skills of researchers and institutions based in the area(s) of study? And how was funding used to improve research infrastructure in the area of study?

   Potentially effective investments into long-term skills and opportunities within institutions could include training or mentorship in analytical techniques and manuscript writing, opportunities to lead all or specific aspects of the study, financial remuneration rather than requiring volunteers, and other professional development and educational opportunities.

   Improvements to research infrastructure could be funding of extended trial designs (such as platform trials) and use of master protocols to enable these designs, establishment of long-term contracts for research staff, building research facilities, and local control of funding allocation.

**Skills:**
Funding for this study supported LBM towards her doctoral degree in Basel, Switzerland. LBM, TM, NN, JM and SK also received financial support to attend conferences and train in the analytic methods required to complete this research.
**Research infrastructure:**
Improvements to research infrastructure in Switzerland were not an explicit outcome of this study.

4. How did you safeguard the researchers who implemented the study?

Please describe how you guaranteed safe working conditions for study staff, including provision of appropriate personal protective equipment, protection from violence, and prevention of overworking.

LBM, JM, TM, NN, SK, and MAP were employed by the Swiss Tropical and Public Health Institute for the duration of this study. The Swiss Tropical and Public Health Institute provides safe working conditions for its researchers in compliance with Swiss employment legislation. This includes a zero-tolerance policy for violence, harassment, and discrimination. All authors and the principle investigator on the funding were encouraged to prevent overworking by setting reasonable deadlines for work towards this study.

<u>Benefits to the communities and regions of study</u>

5. How does the study address the research and policy priorities of its location?

How were the local priorities determined and then used to inform the research question? Who decided which priorities to take forward? Which elements of the study address those priorities?

While based in Switzerland, this study was situated within the global context of research, development and regulation for malaria prevention drugs. As described above, our research questions were developed in response to engagement with this global community as part of the 2021 convening 'Malaria Prevention: Shaping Next-Gen Medical Interventions'. A number of priority research questions were identified as part of the convening and the study team decided which to prioritise for this study of seasonal malaria chemoprevention. Other research questions raised during this convening are being addressed in other work led by LBM, JM, NN, SK, and MAP, co-authored together with other participants from the 2021 convening.

6. How will research products be shared in the community of study?

For instance, will you be providing written or oral layperson summaries for non-academic information sharing? Will study data be made available to institutions in the region(s) of study? The Lancet Global Health *encourages authors to translate the summary (abstract) into relevant languages after paper editing; do you intend to translate your summary?*

Research products have been shared through presentations to key stakeholders in the malaria medical product development community, including drug developers, funders, researchers, and regulators.

7. How were individuals, communities, and environments protected from harm?

a) How did you ensure that sensitive patient data was handled safely and respectfully? Was there any potential for stigma or discrimination against participants arising from any of the procedures or outcomes of the study?

No patient data was collected or handled as part of this study.

b) Might any of the tests be experienced as invasive or culturally insensitive?

As above, no tests were performed as part of this study.

c) *How did you determine that work was sensitive to traditions, restrictions, and considerations of all cultural and religious groups in the study population?*

As above, no patient data was collected or handled as part of this study.

d) *Were biowaste and radioactive waste disposed of in accordance with local laws?*

As above, no biowaste or radioactive waste was generated as part of this study.

e) *Were any structures built that would have impacted members of the community or the environment (such as handwashing facilities in a public space)? If so, how did you ensure that you had appropriate community buy-in?*

As above, no structures were built as part of this study.

f) *How might the study have impacted existing health-care resources (such as staff workloads, use of equipment that is typically employed elsewhere, or reallocation of public funds)?*

As above, no health-care resources were used as part of this study.

8. Finally, please provide the title (eg, Dr/Prof, Mr/Mrs/Ms/Mx), name, and email address of an author who can be contacted about this statement. This can be the corresponding author.

Name: Prof Melissa Penny
Email: melissa.penny@unibas.ch
